# Lipidomic and transcriptomic analysis of western diet-induced nonalcoholic steatohepatitis (NASH) in female *Ldlr ^-/-^* mice

**DOI:** 10.1371/journal.pone.0214387

**Published:** 2019-04-03

**Authors:** Manuel Garcia-Jaramillo, Melinda H. Spooner, Christiane V. Löhr, Carmen P. Wong, Weijian Zhang, Donald B. Jump

**Affiliations:** 1 The Nutrition Program, School of Biological and Population Health Sciences, Oregon State University, Corvallis, Oregon, United States of America; 2 The Linus Pauling Institute, Oregon State University, Corvallis, Oregon, United States of America; 3 Department of Chemistry Oregon State University, Corvallis, Oregon, United States of America; 4 Anatomic Pathology, Carlson College of Veterinary Medicine, Oregon State University, Corvallis, Oregon, United States of America; INRA, FRANCE

## Abstract

**Background:**

Nonalcoholic fatty liver disease (**NAFLD**) is the most common chronic liver disease worldwide, particularly in obese and type 2 diabetic individuals. NAFLD ranges in severity from benign steatosis to nonalcoholic steatohepatitis (**NASH**); and NASH can progress to cirrhosis, primary hepatocellular carcinoma (**HCC**) and liver failure. As such, NAFLD has emerged as a major public health concern. Herein, we used a lipidomic and transcriptomic approach to identify lipid markers associated with western diet (**WD**) induced NASH in female mice.

**Methods:**

Female mice (low-density lipoprotein receptor null (***Ldlr*** ^***-/-***^) were fed a reference or WD diet for 38 and 46 weeks. Transcriptomic and lipidomic approaches, coupled with statistical analyses, were used to identify associations between major NASH markers and transcriptomic & lipidomic markers.

**Results:**

The WD induced all major hallmarks of NASH in female *Ldlr* ^*-/-*^ mice, including steatosis (SFA, MUFA, MUFA-containing di- and triacylglycerols), inflammation (*TNFα)*, oxidative stress *(Ncf2)*, and fibrosis *(Col1A)*. The WD also increased transcripts associated with membrane remodeling *(LpCat)*, apoptosis & autophagy (*Casp1*, *CtsS*), hedgehog (*Taz*) & notch signaling (*Hey1*), epithelial-mesenchymal transition (S1004A) and cancer (*Gpc3*). WD feeding, however, suppressed the expression of the hedgehog inhibitory protein (*Hhip*), and enzymes involved in triglyceride catabolism (*Tgh/Ces3*, *Ces1g)*, as well as the hepatic abundance of C_18-22_ PUFA-containing phosphoglycerolipids (GpCho, GpEtn, GpSer, GpIns). WD feeding also increased hepatic cyclooxygenase (Cox1 & 2) expression and pro-inflammatory ω6 PUFA-derived oxylipins (PGE2), as well as lipid markers of oxidative stress (8-iso-PGF2α). The WD suppressed the hepatic abundance of reparative oxylipins (19, 20-DiHDPA) as well as the expression of enzymes involved in fatty epoxide metabolism (*Cyp2C*, *Ephx*).

**Conclusion:**

WD-induced NASH in female *Ldlr* ^*-/*-^ mice was characterized by a massive increase in hepatic neutral and membrane lipids containing SFA and MUFA and a loss of C_18-22_ PUFA-containing membrane lipids. Moreover, the WD increased hepatic pro-inflammatory oxylipins and suppressed the hepatic abundance of reparative oxylipins. Such global changes in the type and abundance of hepatic lipids likely contributes to tissue remodeling and NASH severity.

## Introduction

Nonalcoholic fatty liver disease (**NAFLD**) is defined as excessive neutral lipid (triglycerides and cholesterol esters) deposition in the liver, i.e., hepatosteatosis [1, 2]. The top 4 risk factors for NAFLD are obesity, dyslipidemia, type 2 diabetes mellitus (**T2DM**) and metabolic syndrome (**MetS**) [3, 4]. NAFLD is a continuum of diseases ranging from benign steatosis to primary hepatocellular cancer (**HCC**). It is strongly associated with obesity [5, 6]; and it is the most common chronic fatty liver disease worldwide [7]. Approximately 30% of the US population is estimated to have some form of chronic fatty liver disease [8].

Ten to 30% of NAFLD patients develop nonalcoholic steatohepatitis (**NASH**), the progressive form of the disease. NASH patients have hepatic steatosis plus hepatic and systemic inflammation, oxidative stress and liver injury [9]. Excessive liver damage resulting from NASH activates tissue remodeling mechanisms involving deposition of extracellular matrix components (**ECM**), leading to fibrosis. There is a high prevalence of NASH (≥60%) in the T2DM population [10]; and NASH is a risk factor for cardiovascular disease [11–13]. NASH patients also have higher mortality rates than NAFLD patients; and both have higher mortality rates than the general population. Twenty to 30% of NASH patients progress to cirrhosis. Over a 10 year period, cirrhosis and liver related deaths occur in 20% and 12% of NASH patients, respectively [9]. By the year 2020, cirrhosis resulting from NASH is projected to be the leading cause of liver transplantation in the United States [14]. Given the increasing prevalence of NASH and its adverse clinical outcomes, NASH is considered a major public health problem [15].

While NAFLD appears to be more prevalent in men than in women [16, 17], others report that lean patients with NAFLD are more frequently female [18]. In fact, data from nearly 700 patients from the NASH Clinical Research Network showed that biopsy proven NASH patients were more likely female by a ratio of 2:1, female to male [19]. Despite the occurrence of NAFLD in human males and females, preclinical NAFLD models typically use male rodents [20]. By way of illustrating this point, we examined 100 recent preclinical NAFLD publications appearing in PubMed using the query terms (NAFLD, mice). Eighty nine percent of publications used male rodents; typically mice. One explanation for this gender bias is that female mice on the C57BL/6J background, a common mouse model for diet-induced obesity, display some resistance to high fat diet-induced obesity [21, 22]. However, multiple factors contribute to gender-specific responses to high fat diets, including diet composition, gonadal hormone status, animal age and X-chromosomal dosage [20, 23–26].

Our goal in this study was to use a lipidomic and transcriptomic approach to identify lipid markers of western diet (**WD**) induced NASH in female low-density lipoprotein receptor null (***Ldlr*** ^***-/-***^) mice. We and others previously established that *Ldlr* ^*-/-*^ mice are particularly prone to WD-induced NAFLD [27–32]. The WD reflects a modern, but unhealthy human diet [33]; it is moderately high in saturated fat and trans-fat (43% total calories), simple sugar (29% total calories), and cholesterol (0.15% w/w) [29]. The moderately high dietary cholesterol coupled with the ablation of the LDL receptor leads to hypercholesterolemia (dyslipidemia) and vascular inflammation, i.e., atherosclerosis. Moreover, the high saturated & trans-fat, plus the high sucrose contribute to the obese phenotype. Finally, the WD is sufficient in essential fatty acids (**EFA**), but the EFA content is low relative to SFA and MUFA. Feeding male *Ldlr* ^*-/-*^ mice the WD results in a reduction in hepatic C_18-22_ ω3 and ω6 PUFA, a phenomenon seen in human NASH [29–31, 34]. Particularly relevant is the finding that male *Ldlr* ^*-/-*^ mice fed the **WD** results in a NASH phenotype that recapitulates many of the phenotypic features seen in human NASH patients, including obesity, dyslipidemia, hyperglycemia, hepatic damage, hepatosteatosis, and the induction of multiple markers of inflammation, oxidative stress and fibrosis [29, 31].

The outcome of our studies provides novel insight into the broad effects of the WD on hepatic gene expression and lipid metabolism. We report major changes in the acyl chain content of neutral & membrane lipids, as well as, the type and abundance of pro-inflammatory and anti-inflammatory/reparative oxylipins. We used an unbiased statistical approach to identify associations between specific lipids and markers for steatosis, inflammation, oxidative stress, fibrosis, notch signaling, epithelial-mesenchymal transition (**EMT**) and cancer. The overall outcome of the analysis identified global changes in lipid metabolism as likely contributors to hepatic lipotoxicity and NASH progression.

## Materials and methods

### Animals and diets

This study was carried out in strict accordance with the recommendations in the Guide for the Care and Use of Laboratory Animals of the National Institutes of Health. All procedures for the use and care of animals for laboratory research were approved by the Institutional Animal Care and Use Committee at Oregon State University (Permit Number: A3229-01). Female *Ldlr* ^*-/-*^ mice [B6; 129S7-*Ldlr*^*tm1He*r^/J, stock# 002207, purchased from Jackson Labs] were group housed (4 mice/cage) and maintained on a 12-hour light/dark cycle. Mice were acclimatized to the OSU animal facilities for 2-weeks before proceeding with experiments.

**Fig 1:**
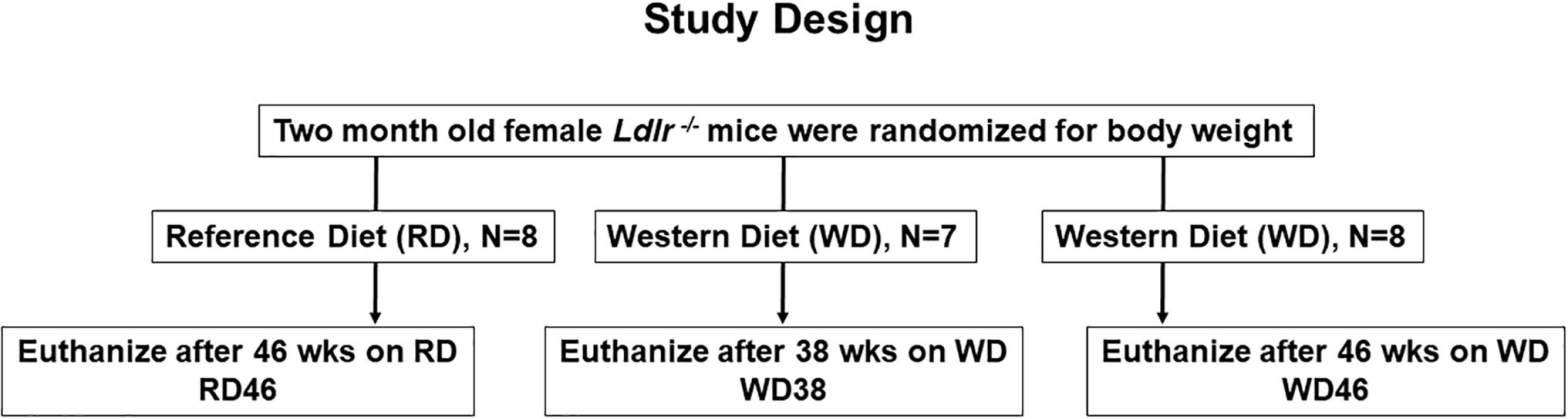
Study design.

At 2 months of age female mice were randomized to 2 treatment groups; 8 mice were maintained on Purina Pico Lab Diet 5053 *ad libitum* for 46 wks [(Reference Diet, (**RD46)**], while 15 mice were fed the Western Diet (**WD**, Research Diets, D12079B) *ad libitum* for 38 or 46 wks (Fig 1)[31]. The reference diet (RD) (Purina chow 5053) consisted of 13.5% energy as fat, 58.0% energy as carbohydrates, and 28.5% energy as protein [29]. The WD (D12079B; Research Diets) consisted of 41% energy as fat, 43% energy as carbohydrate, 17% energy as protein, and 0.15% w/w cholesterol [29].

A power calculation (http://www.dssresearch.com/toolkit/spcalc/power_a1.asp) was carried out with the following parameters: difference between the test (test value = 8) and control (control value = 4), i.e., mean difference is 2-fold; standard deviation 20% of the mean; 95% confidence, the statistical power for 4 and 6 animals (sample size) was 99.1% and 99.9%, respectively. At the termination of the study, all mice were fasted overnight (6:00 PM to 8:00 AM) prior to euthanasia by CO_2_ administration and exsanguination; blood and liver were collected as previously described [31].

### Plasma and hepatic measures

Plasma glucose, triglycerides, total and free cholesterol were measured using kits obtained from Wako. Plasma non-esterified fatty acids (**NEFA**) and β-hydroxybutyrate (**β-HB**) were measured using kits from Sigma-Aldrich and Zen-Bio, respectively. Plasma aspartate amino transferase **(AST)** and alanine amino transferase **(ALT)** were measured using kits from Thermo-Fischer (Waltham, MA) [29, 31].

### Liver histology

Liver (~100 mg) was fixed in buffered-formalin, paraffin embedded, sliced, and stained with hematoxylin-eosin and sirius red (Nationwide Histology, Veradale, WA) [31]. Each slide contained 2–4 slices/liver. Histological analysis and scoring was performed by a board certified veterinary pathologist using a scoring system established for mouse models of NAFLD/NASH [35].

Steatosis was analyzed as macrovesicular (displacing nucleus) and microvesicular vacuolation and severity was scored as the percentage of affected surface area at 100x using the following scale: **0** (0%), **1** (>5% but <33%), **2** (>33% but <66%), **3** (>66%). Steatosis was objectively quantified as percent surface area occupied by lipid vacuoles by image analysis using Image J (NIH; imagej.nih.gov/ij/index.html). Images were taken at 100x from the subjectively least and most severely affected areas. Measurements are presented as the average over two analyzed images per liver normalized to the average percent surface area occupied by lipid vacuoles in liver sections from control mice.

Inflammation was scored as intra-lobular inflammatory foci of at least 5 leukocytes associated with disruption of hepatic plates or hepatocellular organization. Scores are presented as cell count in 3.1 mm^2^ averaged over 5 consecutive fields examined at 40x. The following scoring scale was used: normal (<0.5), slight (>0.5–1), moderate (1–2), severe (>2).

Fibrosis was scored at 40x as absent, mild, moderate or severe. Distribution of fibrosis was recorded as perisinusoidal (s), periportal (p), pericentral (c), and bridging (b). Fibrosis was objectively quantified as percent surface area occupied by sirius red-stained collagen by image analysis using Image J. Images were taken at 100x from the subjectively least and most severely affected areas. Measurements are presented as the average over two analyzed images per liver normalized to the average percent surface area occupied by collagen in liver sections from control mice. Representative images of hepatic micro- and macro-steatosis, leukocyte infiltration and fibrosis are shown in Supplemental Information (S1 and S2 Figs).

### RNA extraction and qRT-PCR

Liver RNA was extracted using Trizol (Life Technologies) and quantified using a Beckman spectrometer at 260 and 280 nm [31, 36]. qRT-PCR was performed using the 7900HT fast machine from Applied Bio-systems as previously described [31, 36]. S1 Table list all transcripts examined in this study and the corresponding primer pairs used for qRT-PCR. In addition, qRT-PCR arrays were used to profile the expression of genes involved in cell death (Mouse Cell Death RT^2^ Profiler PCR Array) according to the manufacturer’s protocol (Qiagen, Valencia, CA, USA). Diet affected genes identified by the Qiagen array analysis were further characterized using in-house generated primer pairs and qRTPCR. S1 Table identifies these transcripts with an (*). Primers were designed using OligoPerfect Primer Designer (Thermo-Fisher, Waltham, MA). Gene expression analysis in the Qiagen arrays was normalized to Hsp90ab1, while cyclophilin was used as the reference gene in the qRTPCR using “in-house” designed primers (S1 Table). Relative quantification was determined using the delta (Δ) Ct method with cyclophilin as the reference transcript [31]. The ΔCt values were used for all statistical analyses. Diet effects on transcript abundance was graphically presented as Fold Change, Mean + SEM, N = 7–8 per group. The statistical package in MetaboAnalyst 4.0 [http://www.metaboanalyst.ca/MetaboAnalyst/] [37, 38] was used for statistical analysis of the transcriptomic data. Accordingly, ANOVA with Tukey’s HSD Post-hoc test was used to identify features that were significantly different amongst the 3 groups: RD46, WD35, WD46. A false discovery rate (**FDR**) value of < 0.05 was the criteria for significance.

### Hepatic lipid composition

Total hepatic lipids were extracted, saponified and the fatty acids converted to fatty acid methyl esters (FAMES) as described [31, 39]. FAMES were separated and quantified by gas chromatography (**GC**) [31, 39]. GC FAMES standards were purchased from Nu-Chek Prep Inc (Elysian, MN). Hepatic protein content was measured using Pierce BCA Protein Assay Kit with bovine serum albumin (BSA) (Thermo-Fisher, Waltham, MA) as a standard. Levels of hepatic fatty acids were normalized to hepatic protein content.

#### Sample preparation for un-targeted and targeted lipidomics

**Un-targeted lipidomics**. All LC-MS/MS analyses described in this report were carried out at the OSU Mass Spectrometry Center (https://mass-spec.science.oregonstate.edu/). The extraction of lipid for lipidomic analysis was adapted from Choi et al [40]. Accordingly, frozen liver (10 mg) was transferred to a 2 ml pre-filled bead rupture tube containing 300 mg of RNAse- and DNAse-free ceramic beads (1.4 mm) (Thomas Scientific) [40, 41]. One ml of degassed methylene chloride:isopropanol:methanol (25:10:65, v/v/v; -20°C) mixture was added to each tube along with a mixture of deuterated lipids (5 μl) (SPLASH Lipidomics Mix, Avanti Polar Lipids) as internal standards. Samples were homogenized using a Precellys 24-bead-based homogenizer for 2 min at 1350 rpm. Samples were incubated at -20°C for 1 hour and then centrifuged at 13,000 rpm and 4°C for 10 min [40, 41]. Extracts were transferred to glass vials and stored less than 24 h at -80°C before analysis.

**Targeted oxylipidomics and relative quantitation**. Oxylipins were extracted from frozen liver (6 mg). Liver was homogenized in 0.5 ml of 10% methanol as described by others [42, 43]. A 10 μl sample of the homogenate was transferred to a microfuge tube for protein quantification (Pierce BCA Protein Assay Kit; BSA as standard) [31]. Homogenized livers were incubated at room temperature for 1h followed by centrifugation 13,000 rpm at 4°C for 10 min. The supernatant (490 μl) was transferred to a methanol-rinsed 1.5 ml microfuge tube and deuterated internal standards (10 μl) were added to each liver extract. The internal standard solution contained 20 deuterated oxylipins at 5 ng/μl each. Oxylipins were further extracted using Strata-X polymerized solid reverse phase extraction columns (Phenomenex, Torrance, CA) as described [43]. Columns were conditioned with methanol and equilibrated with water. Samples were loaded into the cartridge and washed twice with 500 μl of 10% methanol [43]. Additional methanol washes eluted non-specific hydrophobic chemical species and salts from the sample. Oxylipins were eluted from the column with 100% methanol containing 2% formic acid (1 ml), while lipophilic species that could potentially lead to ion suppression were retained on the column [44]. Lipids were stored in 100% methanol at −80°C to minimize non-enzymatic oxidation and degradation until analysis. Just before analysis, samples were evaporated under a stream of nitrogen, re-dissolved in 50 μl of acetonitrile, and incubated in wet ice for 1 h. After a short centrifugation, samples (40 μl) were transferred to glass vials with inserts for analysis.

As noted above, all samples were spiked with deuterated internal standards [45] allowing for the monitoring of extraction efficiencies, changes in ionization of different classes of oxylipins and data normalization [46]. Accordingly, we considered a detected peak was valid when its peak height was 2.5-fold greater than the background noise. Peaks that do not meet this requirement were disregarded. In order to pair a metabolite with a particular internal standard, we used previously reported criteria [42]. S2 Table list all deuterated standards used in this study and their analyte assignments.

#### Reverse-phase UPLC-TOF-MS/MS analysis for lipids and oxylipins

**Un-targeted lipidomics**. Ultra-high performance liquid chromatography (UHPLC) was performed using a Shimadzu Nexera system (Shimadzu, Columbia, MD) coupled to a Triple time-of-flight (TOF) 5600 mass spectrometer (AB SCIEX, Framingham, MA) [47]. Compounds were separated using a Waters Acquity Ultra Performance Liquid Chromatography (UPLC) CSH C18 column (100 mm length × 2.1 mm id (interior diameter); 1.7 μm particle size) coupled to a Waters Acquity VanGuard CSH C18 pre-column (5 mm × 2.1 mm id; 1.7 μm particle size). The column temperature was held constant at 65°C with an eluent flow rate of 0.6 mL min^-1^ [48]. Samples were kept at 4°C throughout the analysis. Samples (1 μl and 5 μl) were injected for electrospray ionization (ESI) positive and negative modes, respectively. Both positive and negative modes used the same mobile phase: (A) 60:40 v/v acetonitrile:water (LC-MS grade) and (B) 90:10 isopropanol:acetonitrile. However, different mobile phase modifiers were used for positive and negative mode analysis in order to improve lipid coverage [49, 50]. For positive mode, 10 mM ammonium formate and 0.1% formic acid was used, while 10 mM ammonium acetate (Sigma–Aldrich) was used for negative mode. The gradient started at 0 min with 15% (B); 0–2 min 15% (B); 2–2.2 min 30% (B); 2.2–9 min 50% (B); 9–9.3 min 80% (B); 9.3–11.8 min 100% (B); 11.8–14 min 15% (B), at a flow rate of 0.6 ml/min [40]. The mass spectrometer was operated in the information-dependent MS/MS acquisition mode: collision energy = 35 V; collision energy spread = 15 V [47].

The TripleTOF instrument was operated in the information-dependent MS/MS acquisition mode under parameters adapted from Kirkwood, et al. [47]. In summary, TOF MS and TOF MS/MS accumulation times were 0.25 and 0.10 s, respectively. The period cycle time was 0.7 s for both TOF MS and TOF MS/MS. The scan range was 70–1700 m/z for TOF MS and 50–1700 m/z for TOF MS/MS. Ion source gas 1 and 2 and curtain gas were set at 50, 45, and 35, respectively. Nitrogen gas was used in all cases. The temperature of the source was set at 550°C. The ion spray voltage was set at 5.5/-5.5 kV. In order to determine the stability of the instrument during the complete run, as well as biological variance, an equal mixture of all liver samples (n = 24) were injected periodically from the beginning to the end of the batch. Additionally, in order to determine potential carry over from one to another injection, blank samples were also periodically analyzed. Auto calibrations (AB SCIEX calibration solution) were performed every five samples in order to correct for small mass drifts during the acquisition [47].

**Targeted oxy-lipidomics**. The same instrumentation and column explained above were used to implement a pseudo multi-reaction monitoring (MRM) method in negative ion ESI mode for the analysis of oxylipins as adapted from Wang et al [43]. Accordingly, the mobile phase consisted of (**A**) acetonitrile/water/acetic acid (60/40/0.02, v/v) and (**B**) acetonitrile/isopropyl alcohol (50/50, v/v). Gradient conditions were as follows: 0–4.0 min, 0.1–55% B; 4.0–4.5 min, 55–99% B; 4.5–5.0 min, 99% B [43]. The sample injection volume was 10 μl aliquot and the flow rate was 0.5 ml/min. of each sample was injected onto the column. The column temperature was kept at 40°C. All samples were kept at 4°C throughout the analysis. Sixty multi-reaction monitoring (MRM) transitions were achieved by flow injection of pure standards and manual optimization by comparison to literature values. This method contains seven different periods corresponding to elution windows for the different compounds. Inside each window, the quantification of the eluted oxylipins was enhanced, assuring optimal dwell time and sufficient data points per peak [51]. For co-eluting isobars a unique fragment ion was chosen. For example, 8,9-DiHETE (m/z 335→185) and 14,15-DiHETE (m/z 335 → 111), both eluting at 1.47 minutes in our chromatography conditions, were quantified by selecting unequal fragment ions.

#### Data processing and statistical analyses for targeted and un-targeted lipidomics

Raw data from targeted oxylipins analyses was imported into MultiQuant software (AB SCIEX) in order to perform the alignment and integration of the peaks (obtaining peak areas). This software allows for the correction of metabolite intensity with the intensity of the internal standards. Data obtained with MultiQuant was imported into MarkerView software (AB SCIEX) for initial data visualization [52]. Student’s t-tests were performed with GraphPad Prism Software (GraphPad, La Jolla, CA). Raw data from un-targeted lipidomic analysis was imported into Progenesis QI software (Version 2.3, Nonlinear Dynamics, Waters) in order to perform data normalization, feature detection, peak alignment, and peak integration [52]. A repeated measure analysis of variance (ANOVA) was also performed using Progenesis QI software in order to identify significantly alter features [52]. Similarly, we obtained the *q*-values based on the *p*-values in order to adjust for multiple comparisons. The optimized false discovery rate (FDR) approach uses characteristics of the *p*-value distribution to produce a list of *q*-values. We considered annotations with a q-value <0.05, which implies a FDR of 5%. The principal component analysis (PCA) for the annotated and significantly affected compounds by the WD was performed in MetaboAnalyst 4.0 [53]. Log transformation and auto-scaling were applied on the data for normality.

Lipid species were confirmed by validated retention times, MS, MS/MS fragmentation, and isotopic distribution using the LipidBlast [54] and Metlin (The Scripps Research Institute) and the Human Metabolome (HMBD) databases as the reference data bases for comparisons [55]. Peak intensities were normalized using the QC pool sample, and the SPLASH Lipidomics Mix (Avanti Polar Lipids, Alabaster, AL).

The statistical package in MetaboAnalyst 4.0 [http://www.metaboanalyst.ca/MetaboAnalyst/] [37, 38] was used to log transform the numerical data and prepare heat maps, principal component analyses and pattern analyses of all anthropometric, plasma, histologic, lipidomic and transcriptomic data. ANOVA with Tukey’s HSD Post-hoc test was used to identify features that were significantly different amongst the 3 groups: RD46, WD35, WD46. We use a FDR-value < 0.05 as the criteria for significance. Paired t-tests were also performed in MetaboAnalyst, with annotated compounds in the 3 groups (RD46 vs WD35 and RD46 vs WD46).

## Results and discussion

### Study design and overall statistical analysis

Female *Ldlr* ^*-/-*^ mice at 2 months of age were randomized for weight, divided into 3 groups and fed a reference (**RD**) or western (**WD**) diet (Fig 1). RD-fed mice were euthanized 46 wks later (**RD46**), while WD-fed mice were euthanized after 38 (**WD38**) and 46 wks (**WD46**) on the WD. Liver and blood were collected for analysis.

**Fig 2:**
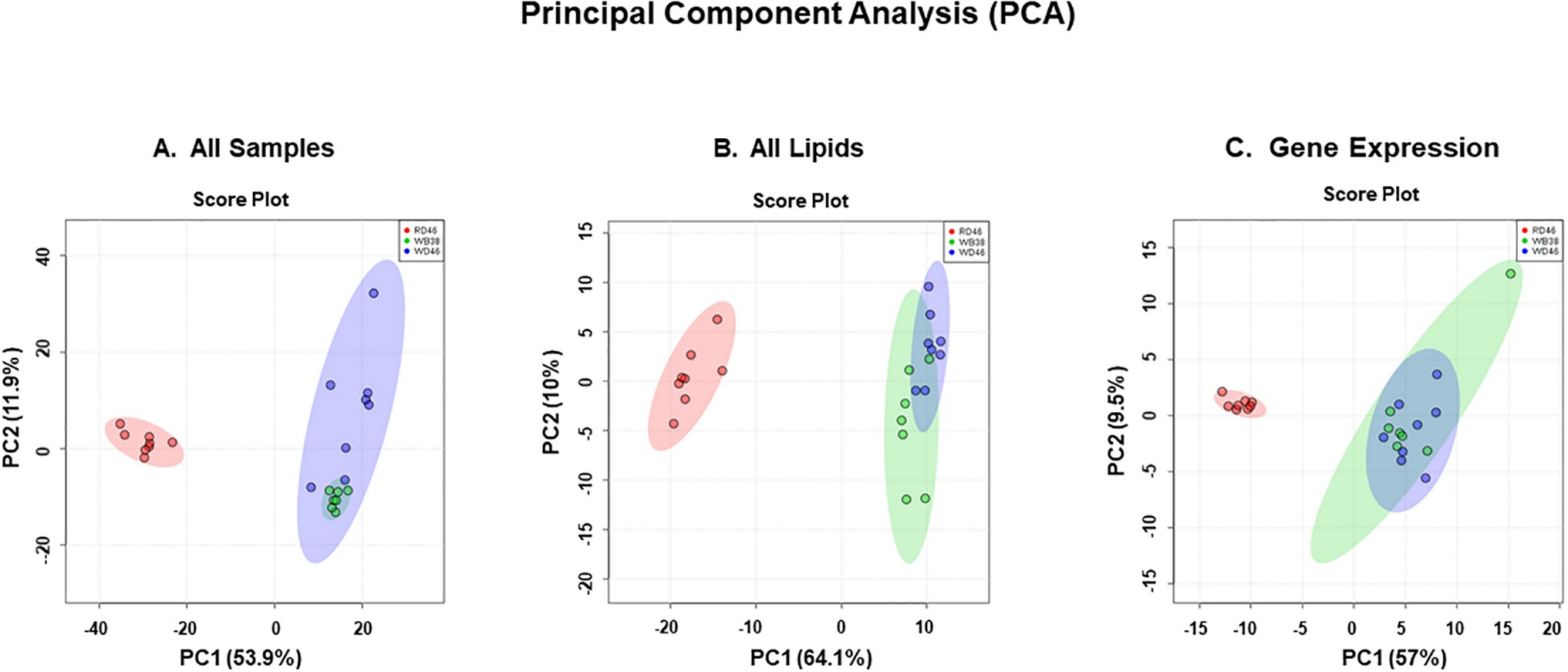
Overall statistical analysis of anthropometric, transcriptomic and lipidomic data. **Panel A:** Principal component analysis (PCA) of all anthropometric, transcriptomic and lipidomic data. All anthropometric, plasma, transcriptomic and lipidomic data was assembled into an Excel spreadsheet for mice in the 3 groups (RD46, WD38 and WD46). The data was analyzed using the statistical package in http://www.metaboanalyst.ca/MetaboAnalyst/ [53]. **Panels B:** PCA analysis of all lipid data including both GC and LC/MS data. **Panel C:** PCA analysis of all transcriptomic data.

We examined anthropometric, plasma and liver histology, gene expression & lipid features [52]. All quantified data was assembled in an excel spreadsheet and analyzed using the statistical package in MetaboAnalyst [31, 37, 38]. Principal component analysis (**PCA**) was used to establish similarities/dissimilarities amongst the 3 groups (Fig 2). Features of mice fed the WD clustered together and were separate from the cluster of the RD-fed mice. As expected, there was overlap in the features of mice fed the WD for 38 and 46 weeks. Separate PCA on only lipid and gene expression markers showed that the two WD groups clustered together and were clearly separate from the RD group (Fig 2 B and 2C). Thus, both lipid and gene expression features contribute to the separation of the RD and WD-fed groups seen in Fig 2A.

### Western diet (WD) induced a NASH phenotype in female *Ldlr* ^*-/-*^ mice

Mice fed the RD for 46 wks had a ~24% increase in body weight over the course of the study (Table 1), while mice fed the WD for 38 or 46 wks increased body weight by 62 and 68%, respectively. Changes in body weight in the WD fed mice represent a 31 and 36% increase in body weight over that seen in the RD-fed mice. WD fed mice also displayed a significant increase in liver weight (LW), i.e., 5.5–5.8 LW%BW, when represented as a percent (%) of body weight (BW) versus that seen in RD mice (3.8 LW%BW). These features were comparable to changes seen in male *Ldlr* ^*-/-*^ mice fed the WD for 16–32 wks [29–31].

**Table 1:** Effect of the western diet on anthropometric, hepatic and plasma features of female *Ldlr* ^*-/-*^ mice^1^.

	RD-46	WD-38	WD-46
**Body & Liver Weight:**			
Animals/group	8	7	8
Initial Body Weight, *g*	17.5 ± 1.0	17.5 ± 1.0	17.4 ± 1.3
Final Body Weight, *g*	21.6 ± 1.6	28.4 ± 4.2*	29.7 ± 4.1*
% Increase Body Weight	123.9 ± 11.8	162.3 ± 24.7*	168.1 ± 20.0*
Liver Weight, *g*	0.8 ± 0.1	1.6 ± 0.5*	1.7 ± 0.4*
LW%BW^2^	3.8 ± 0.3	5.5 ± 0.5*	5.8 ± 1.3*
**Plasma Markers:**			
Glucose, *mg/dl*	210.4 ± 39.9	275.0 ± 109	276.4 ± 53.5
Triglycerides, *mg/dl*	67.3 ± 28.8	81.3 ± 51.9	74.2 ± 24.1
Free Cholesterol, *mg/dl*	46.6 ± 9.5	194.7 ± 75.2*	211.0 ± 78.4*
Total Cholesterol, *mg/dl*	155.1 ± 13.7	498.8 ± 197.4*	482.5 ± 64.0*
NEFA, *μM*	15.9 ± 2.5	20.3 ± 3.1	21.1 ± 4.3*
β-Hydroxybutyrate, *nM*	535.1 ± 144.5	1109.9 ± 385.7*	641.1 ± 236.6
ALT, *U/L*	14.6 ± 2.6	32.4 ± 14.9	44.5 ± 23.9*
AST, *U/L*	13.9 ± 3.8	33.7 ± 15.8*	41.0 ± 1.8*

^1^Results are presented as mean ± SEM, N = 7–8.

Statistical analysis used ANOVA plus Tukey’s HSD to establish statistical significance:

*, p<0.05 versus RD-46 [37, 38, 53].

^2^LW%BW, Liver weight as a % of body weight.

Plasma glucose and triglycerides were not increased by WD feeding. In contrast, free and total plasma cholesterol, NEFA, β-hydroxybutyrate, ALT and AST were elevated in mice fed the WD (Table 1). Increased fasting ALT, AST and cholesterol in WD-fed mice reflects hepatic injury and hypercholesterolemia, respectively. Increased fasting NEFA and β-hydroxybutyrate reflects increased fat mobilization from adipose depots and hepatic ketogenesis, respectively. In contrast to our previous studies with male *Ldlr* ^*-/-*^ mice, the female mice did not develop abnormal plasma triglycerides in response to the WD [29–31].

**Fig 3:**
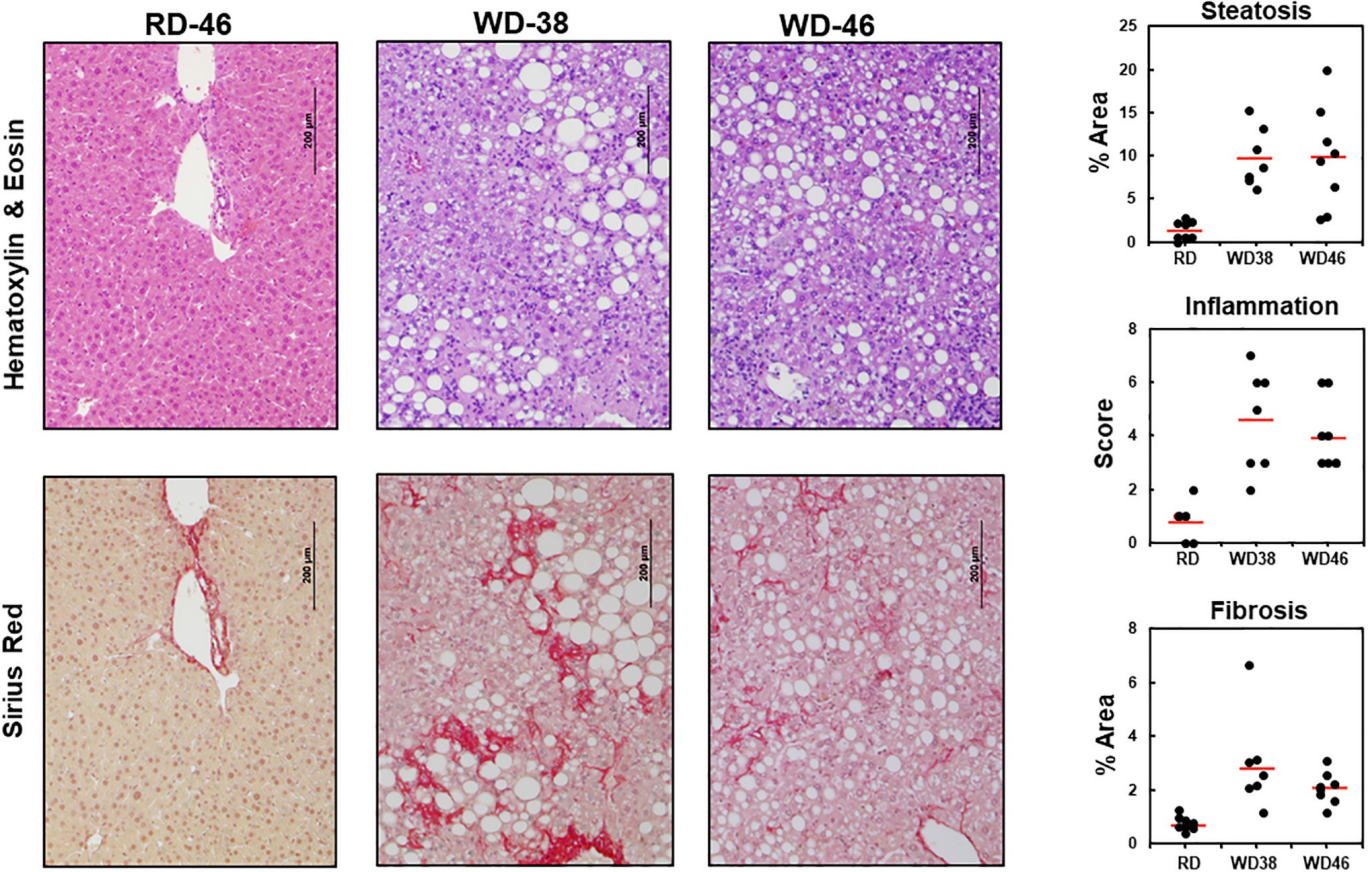
Western diet effects on hepatic morphology. Livers of mice in the 3 groups, RD46, WD38 & WD46, were fixed in buffered formalin, sectioned and stained with hematoxylin and eosin and Sirius red. Images were photographed at 100x; the magnification bar (200 μm) is included on the image. Sections shown are representative of the average (red horizontal line) value for fibrosis in each group. Livers in each group were scored for the level of steatosis, inflammation and fibrosis as described in Materials & Methods and [35]. Data is represented as a scatter plot to illustrate the heterogeneity within each group. Steatosis and Fibrosis are represented as the % Area occupied by lipid droplets and fibrosis, respectively. Inflammation is represented by a score; the scoring scale is: normal (<0.5), slight (0.5–1.0), moderate (1–2), severe (>2). The red horizontal bar represents the average of all animals within each group. Additional histology slides representing micro- and macro-steatosis, inflammation (S1 Fig) and fibrosis (S2 Fig) are included in the Supplement.

Histology of livers from RD and WD-fed mice revealed the major hallmarks of NASH, including micro- and macro-steatosis (hematoxylin/eosin stain) and branching “chicken wire” fibrosis (Sirius red) (Fig 3, S1 and S2 Figs). Quantitation of the histological features described in Materials and Methods revealed heterogeneity in steatosis, inflammation and fibrosis (Fig 3, **right panels**). Heterogeneity in response to the WD was seen in our previous studies with WD-fed male mice [29–31]. This heterogeneity may be linked to variations in the gut microbiome amongst these age-matched mice [56]. Despite this heterogeneity, the majority of WD-fed mice displayed a phenotype characteristic of NASH, i.e., hepatic increased steatosis, leukocyte infiltration of the liver (inflammation) and fibrosis.

We next used a statistical approach to identify highly significant features associated with WD-induced NASH. The top 10 highly significant changes in lipids (FDR, ≤4.4 x 10^−11^) and gene expression (FDR, ≤ 4.4 x 10^−8^) are listed in Table 2. WD consumption suppressed hepatic abundance of lipids containing PUFA (ω3 or ω6) and increased lipids containing SFA and MUFA. This change in lipid type has been reported in patients diagnosed with NASH [29–31, 34]. WD feeding also significantly increased hepatic transcript abundance for enzymes involved in membrane remodeling (*LpCat*), oxidative stress (*Ncf2*), inflammation (*TNFα*), EMT (*S1004A*), Notch (*Hey1*) signaling and apoptosis (*CtsS*), but suppressed hepatic abundance of a transcript encoding an enzyme involved in cholesterol synthesis (*Fdps)*. Suppression of *Fdps* expression is likely linked to the moderately high cholesterol content (0.15% w/w) of the WD.

**Table 2:** Top 10 lipid and gene expression markers of WD-induced NASH in *Ldlr* ^*-/-*^ mice^1^.

**Lipids**	**Fatty Acid Content**	**WD Effect**	**FDR**	**Tukey’s HSD**
ω6 Index	C_18-22_ ω6 PUFA	Decrease	4.4E-14	WD38-RD46; WD46-RD46
ω3 index	C_18-22_ ω3 PUFA	Decrease	7.2E-13	WD38-RD46; WD46-RD46
DG^2^ 34:1	18:1,ω7; 16:0	Increase	7.2E-13	WD38-RD46; WD46-RD46
TG^3^ 51:1	16:0, 17:0, 18:1,ω9	Increase	3.6E-12	WD38-RD46; WD46-RD46
MUFA	C_14-22_ MUFA	Increase	1.3E-11	WD38-RD46; WD46-RD46
18:1,ω9	18:1,ω9	Increase	1.3E-11	WD38-RD46; WD46-RD46
TG 58:3	18:1,ω9; 18:2,ω6, 22:6,ω3	Decrease	1.3E-11	WD38-RD46; WD46-RD46; WD46-WD38
18:0	18:0	Increase	1.3E-11	WD38-RD46; WD46-RD46
GpCho^4^ 36:5	18:3,ω3, 18:2,ω6	Decrease	3.7E-11	WD38-RD46; WD46-RD46
14:0	14:0	Increase	4.4E-11	WD38-RD46; WD46-RD46
**Gene Expression**	**Pathway**	**WD Effect**	**FDR**	**Tukey’s HSD**
LpCat2	Membrane remodeling	Increase	3.7E-11	WD38-RD46; WD46-RD46
Ncf2	Oxidative Stress	Increase	7.1E-10	WD38-RD46; WD46-RD46
TNFα	Inflammation	Increase	1.3E-09	WD38-RD46; WD46-RD46
S100A4	EMT^5^	Increase	1.4E-09	WD38-RD46; WD46-RD46
Hey1	Notch Signaling	Increase	1.7E-09	WD38-RD46; WD46-RD46
HeyL	Notch Signaling	Increase	2.1E-09	WD38-RD46; WD46-RD46
Fdps	Cholesterol Metabolism	Decrease	6.0E-09	WD38-RD46; WD46-RD46; WD46-WD38
Bcl2	Apoptosis	Increase	8.8E-09	WD38-RD46; WD46-RD46
CtsS	Apoptosis	Increase	9.1E-09	WD38-RD46; WD46-RD46
LpCat1	Membrane remodeling	Increase	4.4E-08	WD38-RD46; WD46-RD46

^1^All data from the anthropometric, plasma, gene expression and lipid analyses was assembled into an excel spreadsheet and analyzed using the statistical package in Metabolanalyst [37, 38, 53]. The results above are based on an ANOVA (one-way) analysis with a post-hoc Tukey’s honest significant difference test (HSD). Statistical significance was based on the false discovery rate (FDR). The results in this table represent the top 10 lipids (Lipid) and gene expression (mRNA) markers affected by the WD.

^2^DG, diacylglycerols;

^3^TG, triacylglycerol,

^4^GpCho, Phosphatidylcholine;

^5^EMT, epithelial mesenchymal transition.

**Fig 4:**
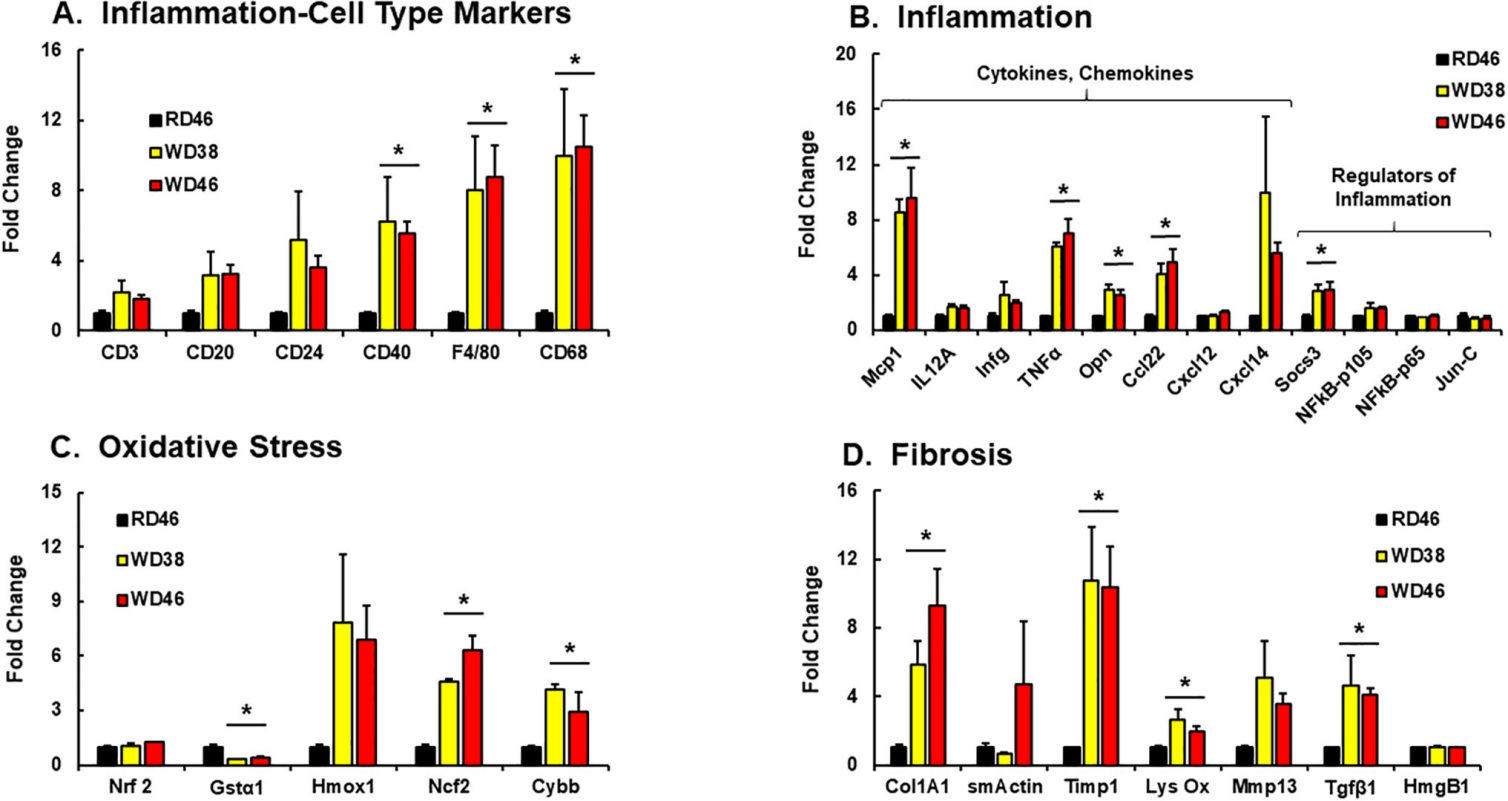
Western diet effects on hepatic gene expression linked to inflammation (A & B), oxidative stress (C) and fibrosis (D). Hepatic transcripts were quantified by qRTPCR using cyclophilin as the reference gene. Transcript (mRNA) abundance is expressed as Fold Change (mean ± SEM, N = 7–8/group); *, FDR ≤ 0.05 versus the RD46 group. [**A**: CD3, T-cell marker; CD20, B-cell marker, CD24, granulocyte and B-cell marker, CD40, TNF-receptor superfamily marker; F4/80, macrophage/Kupffer cell marker; CD68, macrophage/Kupffer cell marker]. [**B**: Mcp1, monocyte chemoattractant protein-1, Il12A, interleukin 12A, Infg, interferon γ; TNFα, tumor necrosis factor α; Opn, osteopontin; Cxcl12, ligand for chemokine C-X-C receptor 4; Cxcl14, ligand for a C-X-C chemokine receptor; Socs3, suppressor of cytokine signaling-3; NFκB-p105, mRNA encoding p50 subunit of NFκB; NFκB-p65, mRNA encoding p65 subunit of NFκB; Jun-C, Ap1 transcription factor]; [**C**: Nrf2, nuclear factor (erythroid-derived 2)-like 2; Gstα1, glutathione S transferase α1; Hmox1, heme-oxygenase 1; Ncf2, neutrophil cytosol factor 2 (p67 phox): Cybb, cytochrome B245, β-peptide (gp91-phos, Nox2)]; [**D**: Col1A1, collagen 1A1; smActin, smooth muscle actin; Timp1, tissue inhibitor metalloprotease-1; Lys Ox, lysyl oxidase; Mmp13, matrix metalloprotease-13; Tgfβ1, transforming growth factor β1; HmgB1, high mobility group protein B1].

**Fig 5:**
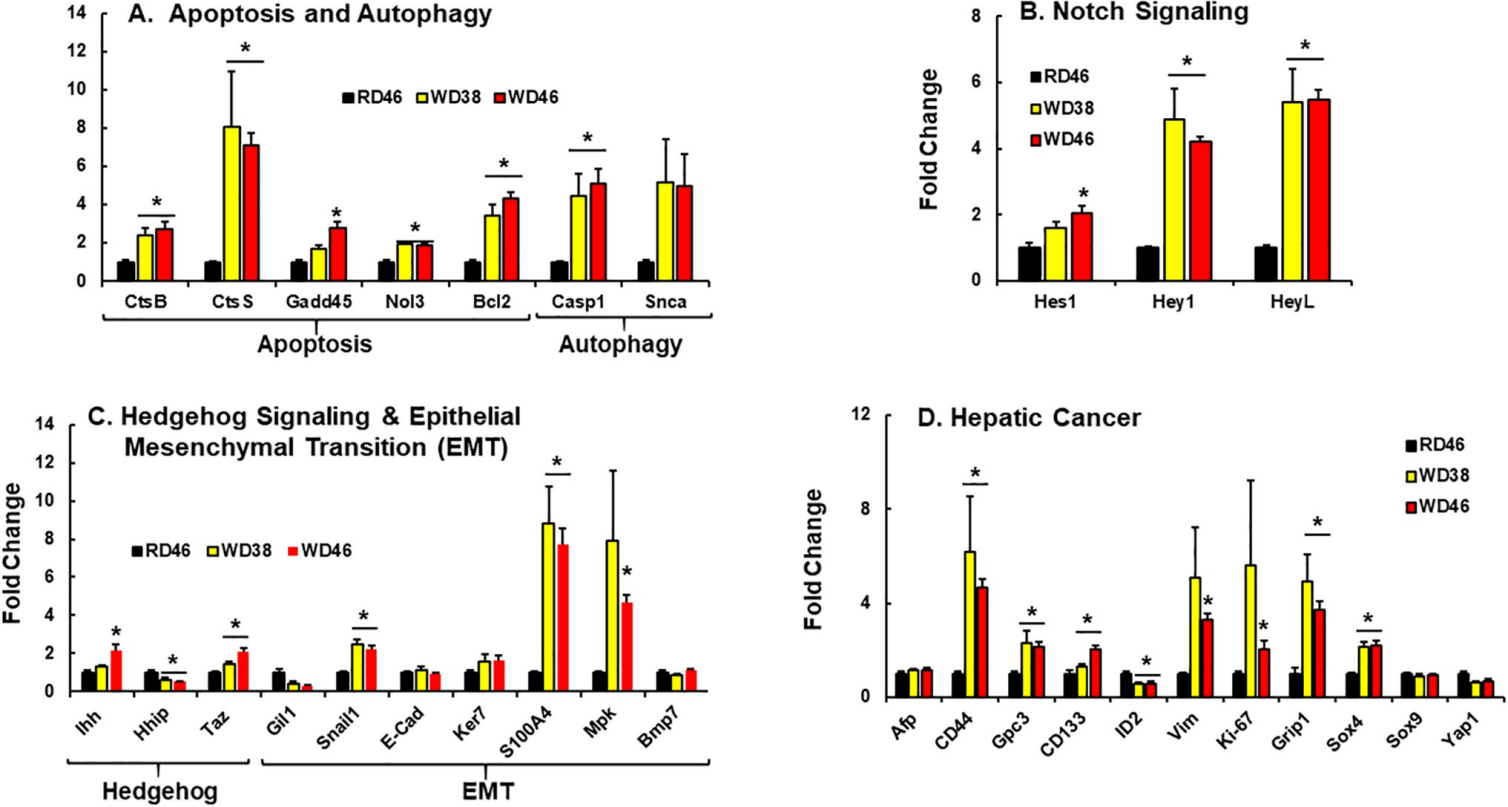
Western diet effects on hepatic gene expression linked to apoptosis and autophagy (A), notch signaling (B); hedgehog and epithelial-mesenchymal transition (EMT) (C) and cancer (C). Hepatic transcripts were quantified by qRTPCR using cyclophilin as the reference gene. Transcript (mRNA) abundance is expressed as fold change (mean ± SEM, N = 7–8/group) *, FDR ≤ 0.05 versus the RD46 group. [**A**: CtsB, cathepsin B; CtsS, cathepsin S; Gadd45, growth arrest and DHA damage 45α; Nol3, nucleolar protein 3; Bcl2, B-cell lymphoma 2; Casp1, caspase 1; Snca, α-synuclein]. [**B**: Hes1, hairy & enhancer of split 1; Hey1, hairy & enhancer of split related with YRPW motif protein 1; HeyL, hairy & enhancer of split related with YRPW motif protein L]. [**C**: Ihh, indian hedgehog; Hhip, hedgehog inhibitory protein; Taz, WW domain containing transcription regulator (WWRT1); Gil1, Glioma-associated protein-Kruppel family member 1; Snail1, snail family zinc finger-1; Ker7, keratin 7; S100A4, S100 calcium binding protein A4; Mpk, muscle pyruvate kinase; Bmp7, bone morphogenic protein 7]. [**D**: Afp, α-fetoprotein; CD44, cluster of differentiation 44; Gpc3, glypican 3; CD133, cluster of differentiation-133; ID2, inhibitor of DNA binding 2; Vim, vimentin; Ki-67, marker of proliferation-67; Grip1, glutamate receptor interacting protein-1; Sox4,SYR (sex determining region Y-box4; Sox9, sex determining region Y-box 9; Yap1, yes-associated protein 1].

Further probing of WD effects on hepatic gene expression focused on inflammation, oxidative stress, fibrosis, apoptosis & autophagy, notch & hedgehog signaling, EMT and cancer (Figs 4 and 5). The WD induced hepatic mRNA abundance of multiple inflammation markers, including markers of macrophage (*F4/80* [*Emr1*], *Cd68*), chemokines (*Mcp1*, *Ccl22*, *Cxcl14*), secreted cytokines (*TNFα*, *Opn*) and the suppressor of cytokine signaling (*Socs3*) (Fig 4A and 4B). The increase in hepatic granulocytes (S1 Fig) is associated with increased hepatic abundance of macrophage markers (*CD68*, *F4/80*) and the production of chemokines (*Mcp1*) and cytokines (*TNFα*), but not B-cell (*CD20*, *CD24*) or T-cell (*CD3*) markers.

The WD induced the hepatic abundance of mRNAs (≥3-fold) encoding two enzymes involved in oxidative stress (Fig 4C), i.e., NADPH-oxidase subunits [Ncf2 (p67phox) and Cybb (cytochrome b245, Gp91 phox, Nox2)]. While Gstα1 mRNA abundance was suppressed (>50%) by WD, Nrf2 mRNA, a major transcriptional regulator of oxidative stress, was not affected by the WD. Oxidative stress is associated with human NAFLD and vitamin E supplementation has been recommended as one approach to combat NAFLD-associated oxidative stress [57, 58]

WD-induced fibrosis (Figs 3 and 4D) was associated with increased expression of several fibrosis markers, including (*Col1A1*, *Timp1*, *Lys Ox*, *Mmp13 and Tgfβ1*). However, not all fibrosis markers were induced by the WD, including *smActin* and *HmgB1* (a transcription factor linked to chronic liver disease and fibrosis [59].

NAFLD is associated with increased apoptosis and autophagy in humans and mice [2, 60, 61]. Female *Ldlr*^*-/-*^ mice fed the WD have elevated hepatic mRNA for key proteins linked to apoptosis (*CtsB*, *CtsS*, *Gadd45*, *Nol3*, *Bcl2*) and autophagy (*Casp1*) (Fig 5A). All markers were induced ≥ 2-fold in mice fed the WD. Since apoptosis and autophagy are associated with cellular and tissue remodeling, we examined WD effects on notch & hedgehog signaling and EMT (Fig 5B and 5C) [62, 63]. Activation of notch signaling is associated with the ≥2-fold induction of key transcription factors i.e., Hes1, Hey1 and HeyL. While the mRNAs encoding indian hedgehog (*Ihh*), a hedgehog ligand, and the transcription factor Taz, a downstream target of hedgehog signaling [64], were induced ≤2-fold by the WD. The mRNA encoding the hedgehog inhibitory protein (*Hhip*), however, was suppressed by 50%. Three of the seven EMT markers (Snail1, S1004A, Mpk) were induced in livers of WD-fed mice. Together, these findings indicate that WD feeding induced hepatic mRNAs encoding proteins involved in apoptosis, autophagy, hedgehog & notch signaling and EMT in female *Ldlr* ^*-/*-^ mice.

Since NASH has the potential to progress to primary hepatocellular cancer (**HCC**) in humans [65, 66], we asked if female *Ldlr* ^*-/-*^ mice displayed evidence of hepatic cancer. Expression of eight of the eleven cancer markers examined (*Cd44*, *Gpc3*, *Cd133*, *Bcl2*, *Vim*, *Ki-67*, *Grip1*, *Sox4*) were affected by the WD [67, 68] (Fig 5D). Some cancer markers (*Afp*, *Sox9*) were not affected by the WD, while one marker, ID2 (inhibitor of DNA binding) was suppressed by 50% in livers of WD-fed mice. Changes in mouse hepatic ID2, Vim, and S100A4 are consistent with findings reported in human HCC [68, 69] and pancreatic cancer [70]. As such, this analysis indicates that female *Ldlr* ^*-/-*^ mice fed the WD for 38 to 46 wks become obese and increase hepatic expression of multiple transcriptomic markers of NASH and HCC.

### The WD significantly alters the hepatic lipidome

**Fig 6:**
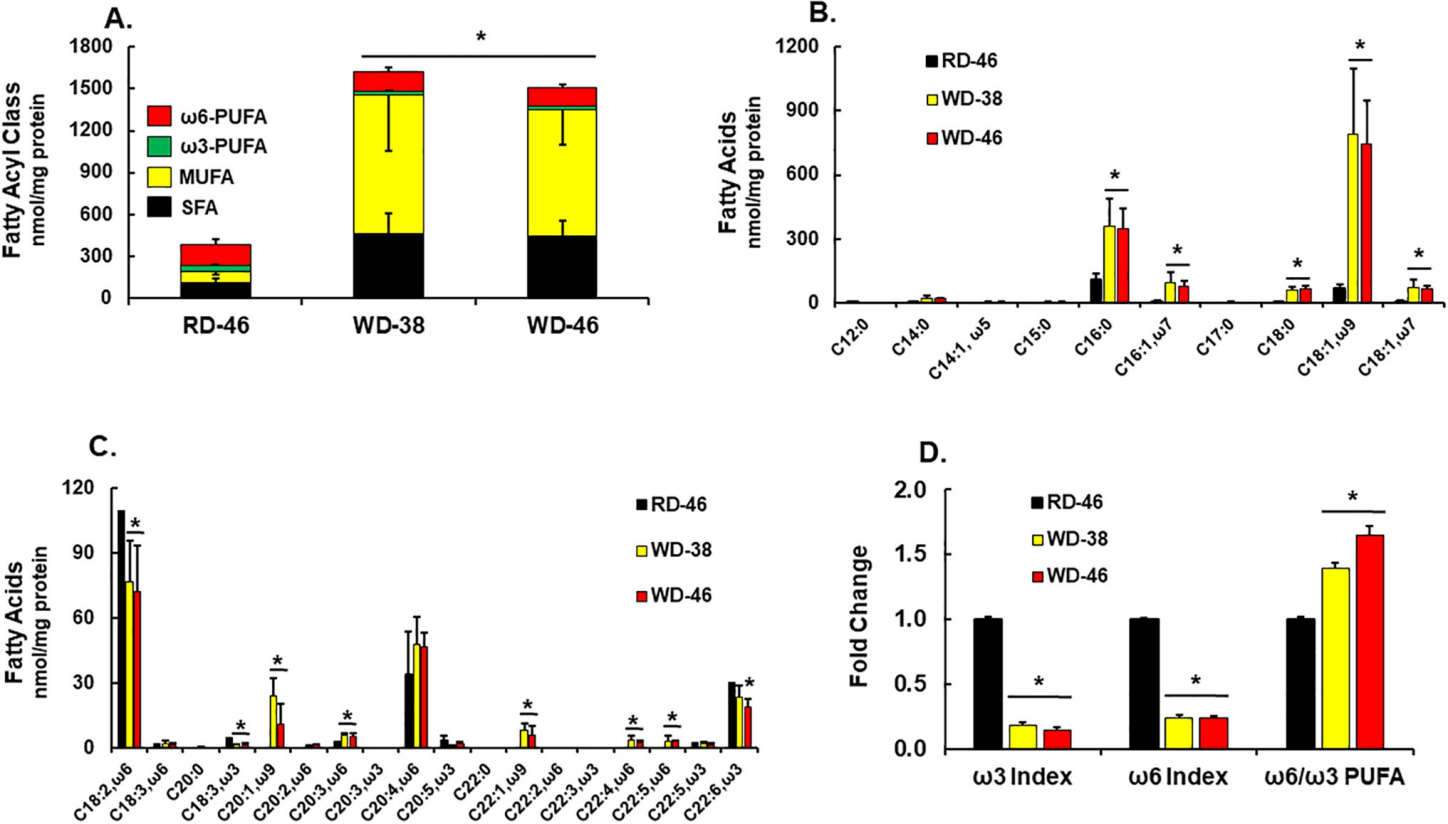
Western diet effects on hepatic fatty acid composition. Livers were extracted for total lipids, saponified and converted to fatty acid methyl esters (FAMES). FAMES were quantified using a gas chromatograph equipped with a flame ionization detector (Materials & methods). Results are expressed as the sum of SFA, MUFA, ω3 & ω6 PUFA, nmoles/mg protein (**A**), specific fatty acyls (**B & C**) and the ω3 index, ω6 index & the ratio of ω6 to ω3 PUFA (**D**). The ω3 and ω6 index represent the fraction of all fatty acids that are either ω3 PUFA or ω6 PUFA. Results in Panel D were normalized to levels in the RD46 group. Results are expressed as mean ± SEM, N = 7–8/group, *, FDR <0.05 versus the RD46 group.

We [29] and others [71] have examined the hepatic lipidome in an effort to identify potential lipid mediators of NALFD. Herein, we use gas chromatographic (GC) and LC-MS/MS (un-targeted and targeted) approaches to identify and quantify hepatic lipids. The GC analysis of free and saponified fatty acids showed that consumption of the WD was associated with a massive increase of hepatic saturated (**SFA**) and monounsaturated fatty acids (**MUFA**) (Fig 6), specifically 16:0; 18:0; 16:1,ω7; 18:1,ω7; 18:1,ω9; 20:1,ω9 & 22:1,ω9. While C_16-18_ SFA and MUFA are in the WD, C_20-22_ SFA and MUFA are not [29]. As such, *in vivo* metabolic pathways account for the increase in C_20-22_ SFA and MUFA.

While the WD is sufficient in essential fatty acids [EFA] (linoleic acid, **LA**, 18:2, ω6; α-linolenic acid, **ALA** 18:3,ω3), the diet abundance of these EFA is low when compared to dietary SFA and MUFA [29]. The WD had minimal effects on hepatic C_18_ ω6 PUFA content and no effect on hepatic arachidonic acid levels (**ARA**; 20:4, ω6); ARA is an elongation & desaturation product of LA, an EFA (Fig 6C). The C_22_ ω6 PUFA (22:4, ω6 and 22:5, ω6) are elongation products of ARA. Since these C_22_ ω6 PUFA are not in the WD [29], these fatty acids increased as a result of ARA elongation & desaturation (Fig 6C). In contrast, WD feeding lowered hepatic ALA and docosahexaenoic acid (**DHA**; 22:6,ω3) by ≤ 35%. Since the WD contains no DHA [29], DHA was derived from ALA through the same desaturation, elongation and peroxisomal β-oxidation pathways as C_22_ ω6 PUFA (Fig 6C). As such, the decline in DHA may be due to decreased substrate (ALA) availability, inhibition of ALA elongation and/or desaturation or competition of SFA or MUFA for enzymes involved in elongation & desaturation [72]. Regardless of the mechanism, the outcome of WD feeding is a selective retention of ARA and an accumulation of its elongation & desaturation products and a loss of ALA and its C_20-22_ elongation & desaturation products. Because of the massive increase in hepatic SFA and MUFA resulting from WD feeding, the relative abundance of ω3 and ω6 PUFA decreased (ω3 & ω6 index) (Fig 6D). Consequently, WD feeding increases C_18-22_ ω6 PUFA (by 65%), relative to C_18-22_ ω3 PUFA (ω6/ω3 PUFA) (Fig 6D).

**Fig 7:**
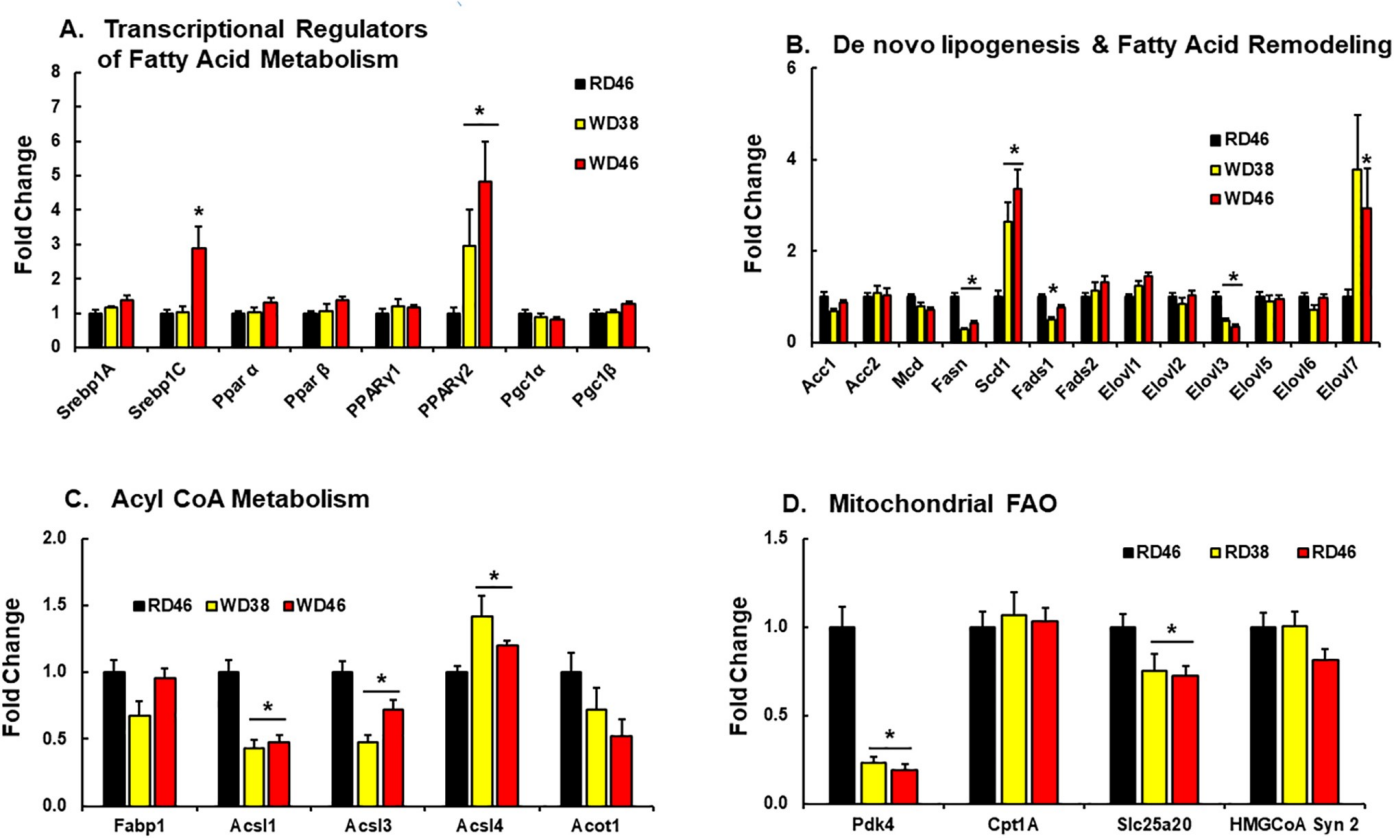
Western diet effects on hepatic gene expression linked to transcriptional regulators of fatty acid metabolism (A), *de novo* lipogenesis & fatty acid remodeling (B), acyl CoA metabolism (C) and mitochondrial fatty acid oxidation (FAO) (D). Hepatic transcripts were quantified by qRTPCR using cyclophilin as the reference gene. Transcript (mRNA) abundance is expressed as fold change (mean ± SEM, N = 7–8/group), *, FDR ≤ 0.05 versus the RD46 group. [**A**: SREBP, sterol regulatory element binding protein; PPAR, peroxisome proliferator activated receptor; PGC, PPARγ co-activator]; [**B**: Acc, acetyl CoA carboxylase; Mcd, malonyl CoA decarboxylase; Fasn, fatty acid synthase; Scd1, stearoyl CoA desaturase; Fads, fatty acid desaturase; Elovl, fatty acid elongase]; [**C**: Fabp, fatty acid binding protein; Acsl, acyl CoA synthetase-long chain; Acot, acyl CoA thioesterase]; [**D**: Pdk4, pyruvate dehydrogenase kinase-4, Cpt1a, liver carnitine palmitoyl transferase 1a; Slc25a20, carnitine acyl-carnitine translocase; Hmg CoA syn 2, HMG CoA synthase 2].

To identify possible mechanisms accounting for WD effects on hepatic fatty acyl content, we quantified the abundance of mRNAs encoding transcription factors regulating hepatic fatty acid metabolism (Fig 7A), as well as enzymes involved fatty acid metabolism (Fig 7B and 7C). All mice in this study were fasted overnight in order to obtain fasting blood samples for the quantitation of blood lipids. Some hepatic transcription factors, like SREBP1c, and enzymes involved in *de novo* lipogenesis (**DNL**) are induced after refeeding fasted mice [28, 73]. However, we examined transcripts in the fasted state only.

Of the genes examined, only *SREBP1c* (at 46 wks) and *PPARγ2* (at 38 & 46 wks) were induced (≤ 2-fold) by the WD. Others have reported that increased hepatic PPARγ2 expression is associated with diet-induced hepatosteatosis [74]. We examined downstream targets of SREBP1c and found no induction of expression of transcripts encoding enzymes involved in malonyl CoA metabolism (*Acc1*, *Acc2*, *Mcd*) or DNL. In fact, expression of *Fasn*, a major DNL enzyme and target of SREBP1c, was suppressed (≤60%) in mice fed the WD. We previously reported that WD-fed male *Ldlr*^*-/-*^ mice express low levels of *Fasn* [29]. Since *Fasn* mRNA parallels changes in hepatic DNL, increased DNL cannot account for the massive increase in SFA or MUFA (Fig 6) [75, 76]. Although the WD has a high sucrose content (~23% total calories, Research Diets), the suppression of *Fasn* expression by the WD suggests that the massive increase in SFA may be more in response to the dietary fat, versus the conversion of dietary carbohydrate to fat through hepatic DNL.

We also quantified diet effects on mRNAs encoding enzymes involved in fatty acid remodeling, i.e., desaturases (*Scd1*, *Fads1*, *Fads2*) & elongases (*Elovl 1–3; Elovl 5–7*). Several of these enzymes (Scd1, Fads1, Fads2) are also downstream targets of SREBP1c, PPARα & PPARγ [73]. While both *Scd1* and *Elovl7* were induced (>2-fold) in livers of WD-fed mice, *Fads1* and *Elovl3* mRNA were suppressed by 50 and ≤65%, respectively. Scd1 is the major hepatic desaturase involved in the MUFA synthesis and both Elovl3 and Elovl7 elongate SFA & MUFA [77]. Increased *Scd1* and *Elovl7* expression likely contribute to the increase in C_20-22_ MUFA (Fig 7B).

Fatty acid binding proteins and fatty acyl CoA formation are requisite events associated with fatty acid trafficking and metabolism. While expression of the major hepatic fatty acid binding protein (*Fabp1*) was unaffected by diet, expression of enzymes involved in acyl CoA formation (*Acsl1 & 3*) were suppressed by ≤50% in livers of WD-fed mice (Fig 7C). This change in gene expression may affect the delivery of substrate to downstream pathways involved in neutral and polar lipid synthesis.

A key pathway for fatty acid disposal is mitochondrial fatty acid β-oxidation (**FAO**). Of the enzymes involved in FAO, i.e., *Pdk4*, *Cpt1A*, *Slc25a20* and *HMG CoA Syn2*, only *Pdk4* mRNA was strongly affected by the WD. Hepatic *Pdk4* mRNA abundance was suppressed by >75% in mice fed the WD (Fig 7D). Insulin is a strong repressor of Pdk4 [78], while PPAR (α, β, γ) activators induce hepatic Pdk4 [79]. Pdk4 plays a key role in substrate selection by controlling pyruvate dehydrogenase activity, a key step for entry of metabolites into the mitochondrial tricarboxylic acid cycle [78, 80]. Attenuated Pdk4 in livers of WD-fed mice may enhance carbohydrate versus fatty acid oxidation.

### Diet effects on neutral and polar lipids

We next examined neutral and polar lipids using LC-MS/MS methods. Among the annotated lipid species identified by time-of-flight (TOF) accurate mass detection and MS/MS fragment characterization, over 90 lipid species were significantly affected by the WD, with *q*-value < 0.05, with an internal coefficient of variation (**CV**) ≤ 30%. The principal component analysis for the annotated lipids significantly affected by the WD (S3 Fig) showed a strong clustering for the 3 treatments, with a clear separation between the RD and WD groups (principal component 1 + principal component 2 > 80%), and overlapping between the mice fed with WD for 38 or 46 wks.

**Fig 8:**
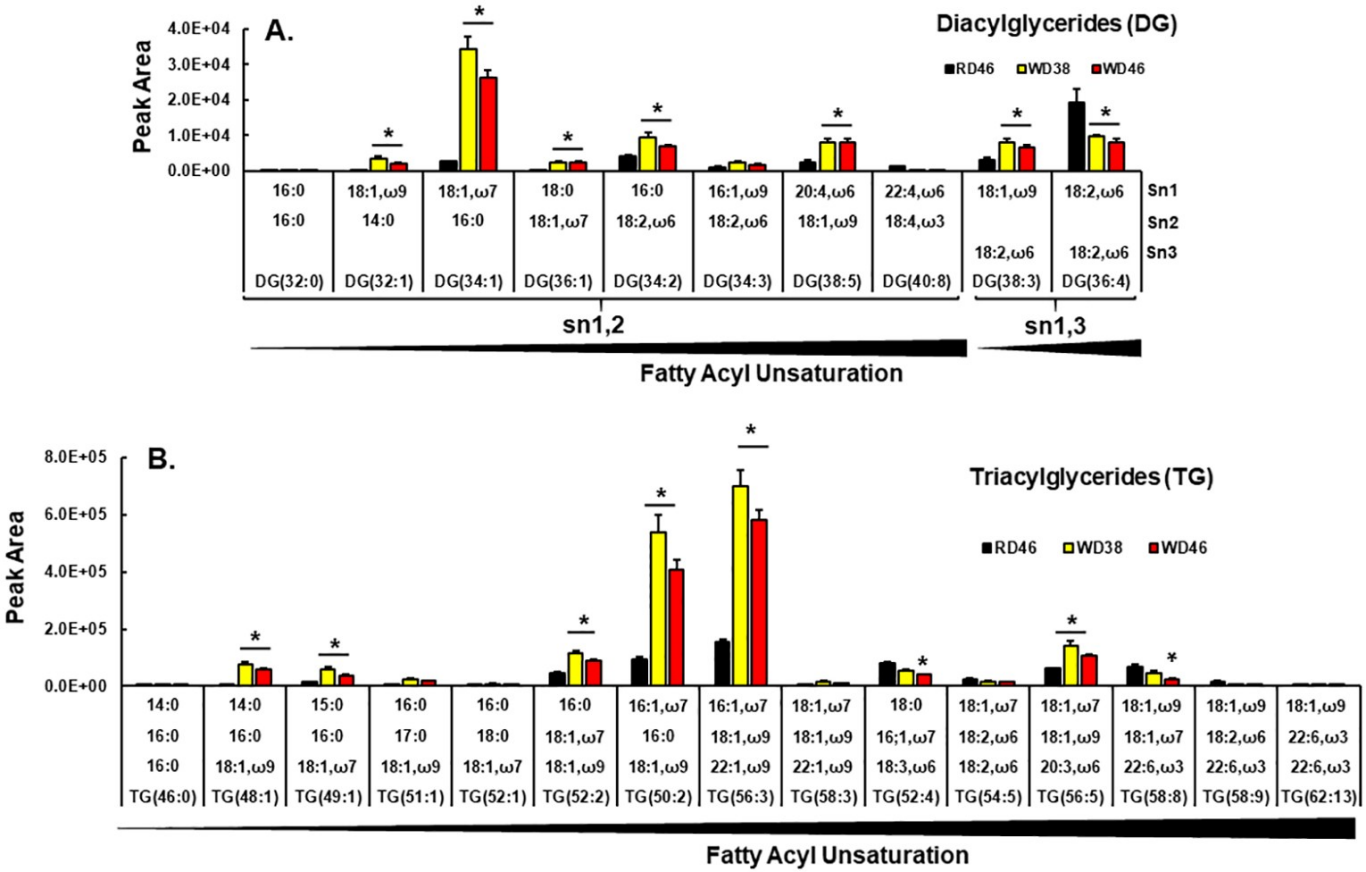
Western diet effects on hepatic diacylglycerols (DG) [A] and triacylglycerols (TG) [B]. DG and TG were quantified using an un-targeted LC/MS approach (Materials and methods). Results in Panel **A** and **B** represents the peak area of specific DG and TG. Results are presented as mean ± SEM, N = 7–8 samples), *, FDR ≤ 0.05 versus the RD46 group. The lipids are arranged by increasing fatty acyl chain unsaturation within specific DG or TG.

**Neutral lipids**. Neutral lipids examined in our analysis include diacylglycerols (**DG**) and triacylglycerols (**TG**). We annotated 10 DG and the relative abundance of 7 DG was significantly affected by the diet (Fig 8A). Those containing SFA and MUFA, i.e., DG 32:1, DG 34:1, DG 36:1, were significantly increased. Two of the 3 DG containing PUFA (DG 34:2, DG 38:5) were significantly increased, while DG 36:4 was significantly decreased in livers of WD-fed mice. Most of the quantified DG had acyl chains in the sn1 & sn2 positions, while two had acyl chains in the sn1 & sn3 positions; sn1,3 DG are generated as a result of TG catabolism, and specifically involve adipocyte triglyceride lipase (ATGL) [81].

Of the 15 annotated TG, 8 were significantly affected by the WD (Fig 8B). As expected, the high abundance of TG in livers of WD-fed mice contained SFA and MUFA, e.g., TG 48:1, TG 49:1; TG 52:2, TG 50:2, TG 56:3. Of the six TG containing PUFA, e.g., TG 52:4, TG 54:5, TG 56:5, TG 58:8, TG 58:9, TG 62:13, only TG 56:5 increased in livers of WD-fed mice. Considering the massive increase of SFA and MUFA in livers of WD fed mice, it is not surprising that the predominant DG and TG were enriched in SFA and MUFA.

**Fig 9:**
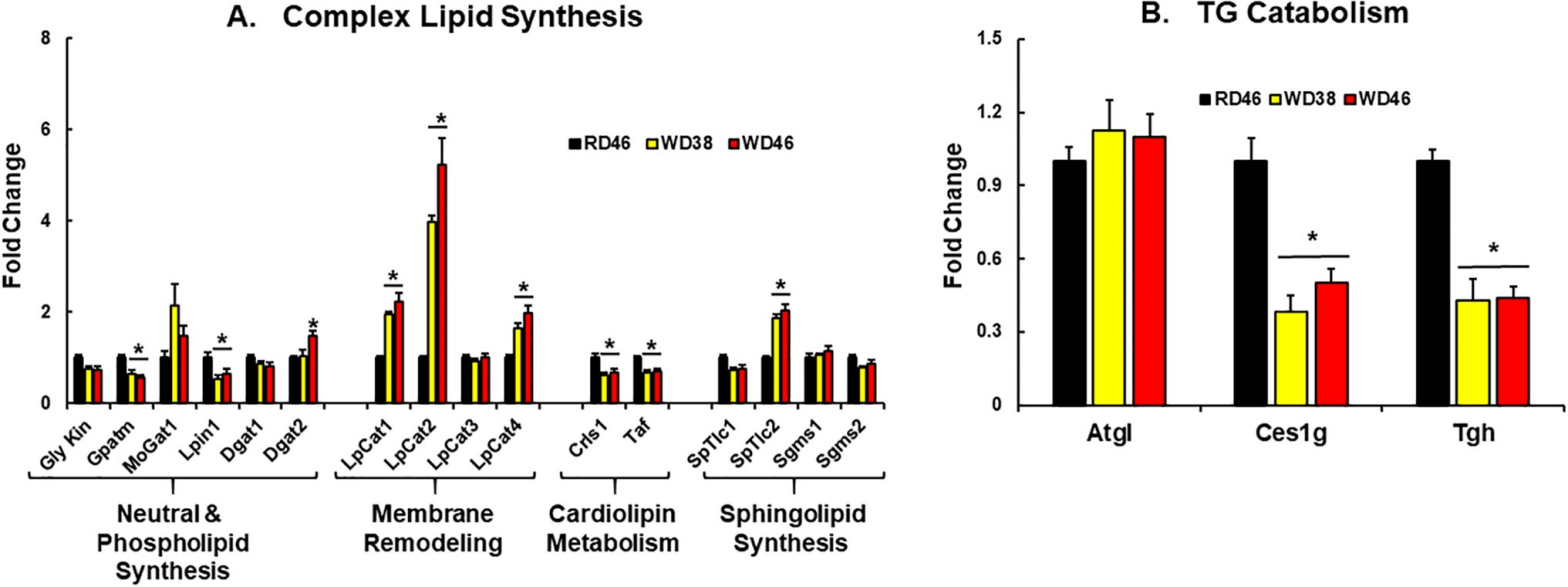
Western diet effects on the expression of enzymes involved in complex lipid synthesis (A) and TG catabolism (B). Transcript abundance was quantified as described above. Results are expressed as mRNA abundance, Fold Change; mean ± SEM, N = 7–8 samples, *, FDR < 0.05 versus the RD46 group. [**A**: Gly Kin, glycerokinase; Gpatm, glycerophosphate acyltransferase-mitochondrial; Dgat, diacylglycerol acyl transferase; LpCat, lysophosphatidyl choline acyltransferase; Crls, cardiolipin synthase; Taf, tafazzin; SpTlc, serine palmitoyl transferase long chain; Sgms, sphingomyelin synthase]; [**B**: Atgl, adipocyte triglyceride lipase, Ces1g, carboxyesterase-1g; Tgh, triglyceride hydrolase/Ces3].

To gain insight into mechanisms mediating changes in neutral lipid composition, we quantified transcripts of enzymes involved in TG synthesis and catabolism (Fig 9). Enzymes involved in DG & TG synthesis include *Gly Kin*, *Gpatm*, *Mogat1*, *Lipin1* and *Dgat 1 & 2* (Fig 9A). The expression of these enzymes changed little in response to the WD. Expression levels of 2 of 3 enzymes involved in neutral lipid catabolism (*Ces1g* and *Tgh)*, however, were reduced by ≤ 50% in livers of WD-fed mice (Fig 9B). Interestingly, ablation of *Tgh* protected mice from diet-induced hepatosteatosis [82], while *Ces1g* deficiency promoted diet-induced fatty liver [83]. TG catabolism is required for assembly and lipidation of ApoB for VLDL assembly and secretion [84, 85]. The lack of fasting hypertriglyceridemia in WD-fed *Ldlr* ^*-/-*^ mice (Table 1) may be an indirect indicator of impaired TG catabolism and/or VLDL assembly & secretion. More studies are required to verify this mechanism.

**Fig 10:**
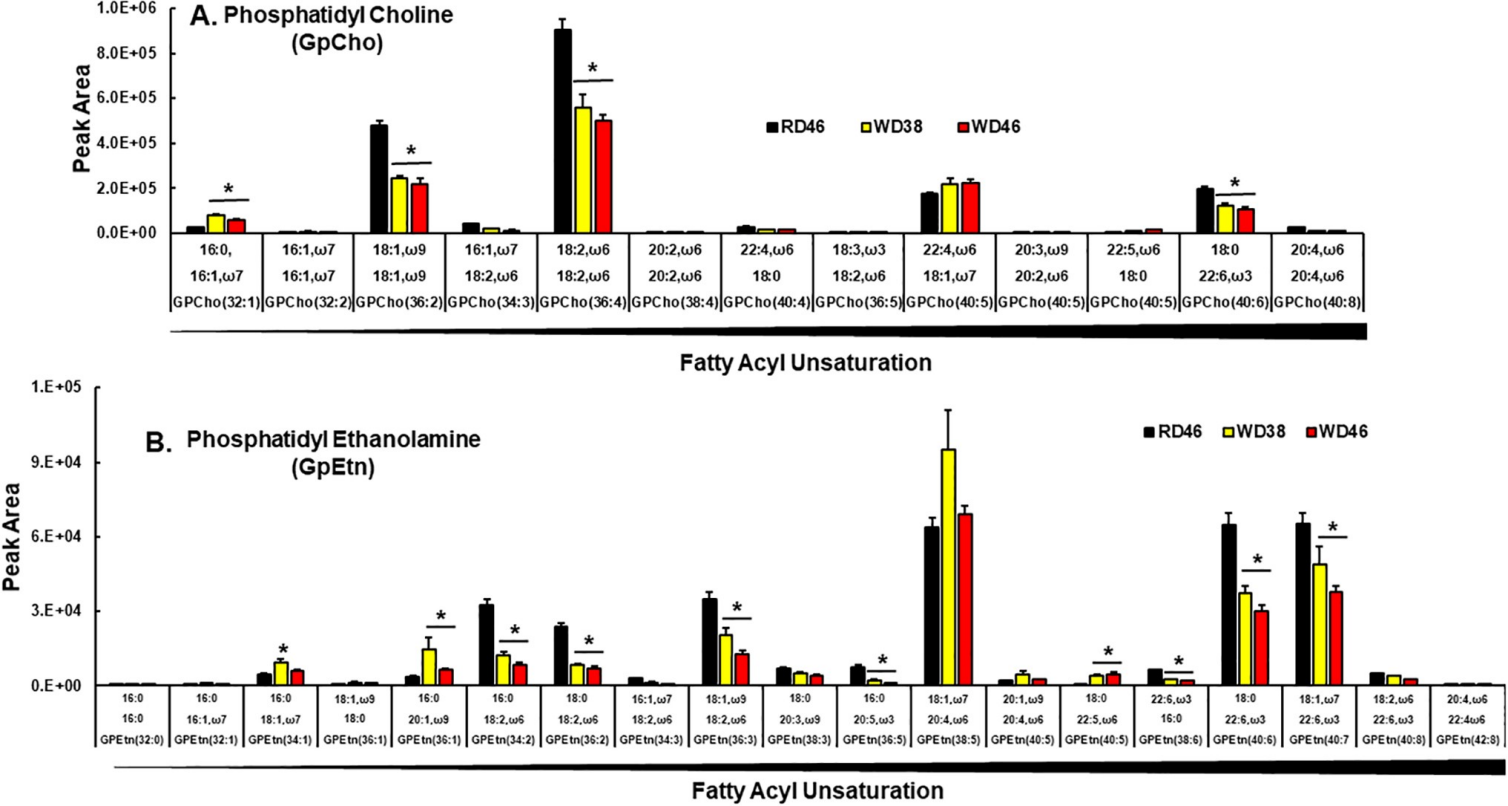
Western diet effects on hepatic phosphatidyl cholines (GpCho) and phosphatidyl ethanolamines (GpEtn). Phosphatidyl cholines (GpCho) were quantified using an un-targeted LC/MS approach (Materials and methods). Results represents the peak area for GpCho (**A**) and GpEtn (**B**) and are presented as mean ± SEM, N = 7–8 samples; *, FDR <0.05 versus the RD46 group. Lipids are arranged as increasing fatty acyl unsaturation within specific glycerophospholipids.

**Polar lipids**. Phosphatidyl cholines (**GpCho**) are prominent lipids in the outer leaflet of the plasma membrane, while phosphatidyl ethanolamines (**GpEtn**), phosphatidyl serines (**GpSer**) and phosphatidyl inositols (**GpIns**) are prominent glycerophospholipids in inner plasma membranes and mitochondrial matrix membranes [86]. Four of the 13 annotated GpCho were affected by the WD (Fig 10A). WD feeding was associated with a ≤ 40% decline in 3 prominent GpCho containing MUFA (GpCho 36:2) and PUFA (GpCho 36:4, GpCho 40:6). Only one GpCho (32:1) increased in response to the WD.

Of the 19 annotated GpEtn annotated (Fig 10B), 2 showed a biphasic (GpEtn 34:1, GpEtn 36:1) response to the WD, while 7 were lower in livers of WD-fed mice. These GpEtn contain PUFA and include GpEtn 34:2; GpEtn 36:2; GpEtn 36:3; GpEtn 36:5; GpEtn 38:6; GpEtn 40:6; GpEtn 40:7). Of the 19 GpEtn examined, only GpEtn 40:5 increased in livers of WD fed mice.

**Fig 11:**
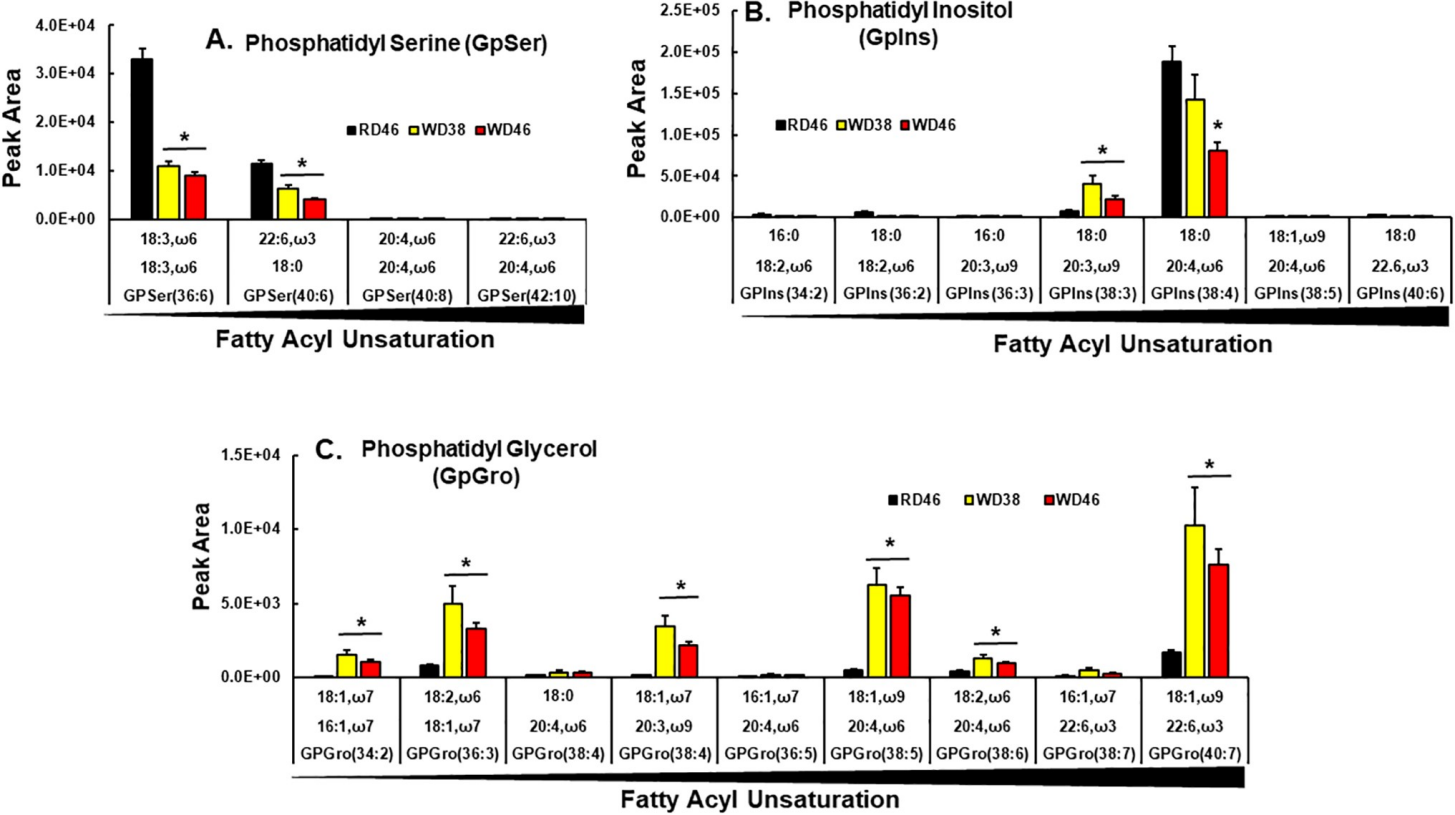
Western diet effects on hepatic phosphatidyl serines (GpSer), phosphatidyl inositols (GpIns) and phosphatidyl glycerols (GpGro). GpSer, GpIns and GpGro were quantified using an un-targeted LC/MS approach (Materials and methods). Results are presented as peak area for GpSer (**A**), GpIns (**B**) and GpGro (**C**), mean ± SEM, N = 7–8 samples; *, FDR <0.05 versus the RD46 group. The results are arranged as increasing fatty acyl unsaturation within specific glycerophospholipids.

Acyl chain composition of 2 (GpSer 36:6, GpSer 40:6) of the 4 annotated GpSer and 2 (GpIns 38:3; GpIns 38:4) of the 7 annotated GpIns was affected by the WD (Fig 11A and 11B). While GpIns 38:3 increased in response to the WD, other GpSer and GpIns were lower (≤ 50%) in livers of WD-fed mice.

Six of the 9 annotated phosphatidyl glycerols (**GpGro**) were affected by the WD, including GpGro 34:2, GpGro 36:3, GpGro 38:4, GpGro 38:5, GpGro 40:7. All but two (GpGro 34:2, GpGro 38:4) contained ω3 or ω6 PUFA (Fig 11C); and all increased in livers of WD-fed mice. GpGro are cardiolipin precursors and cardiolipin is a major mitochondrial matrix phospholipid [87, 88]. Interestingly, increases in mitochondrial ω3 and ω6 PUFA is associated with increased generation of reactive oxygen species (**ROS**) and oxidative stress is linked to NAFLD [89].

**Fig 12:**
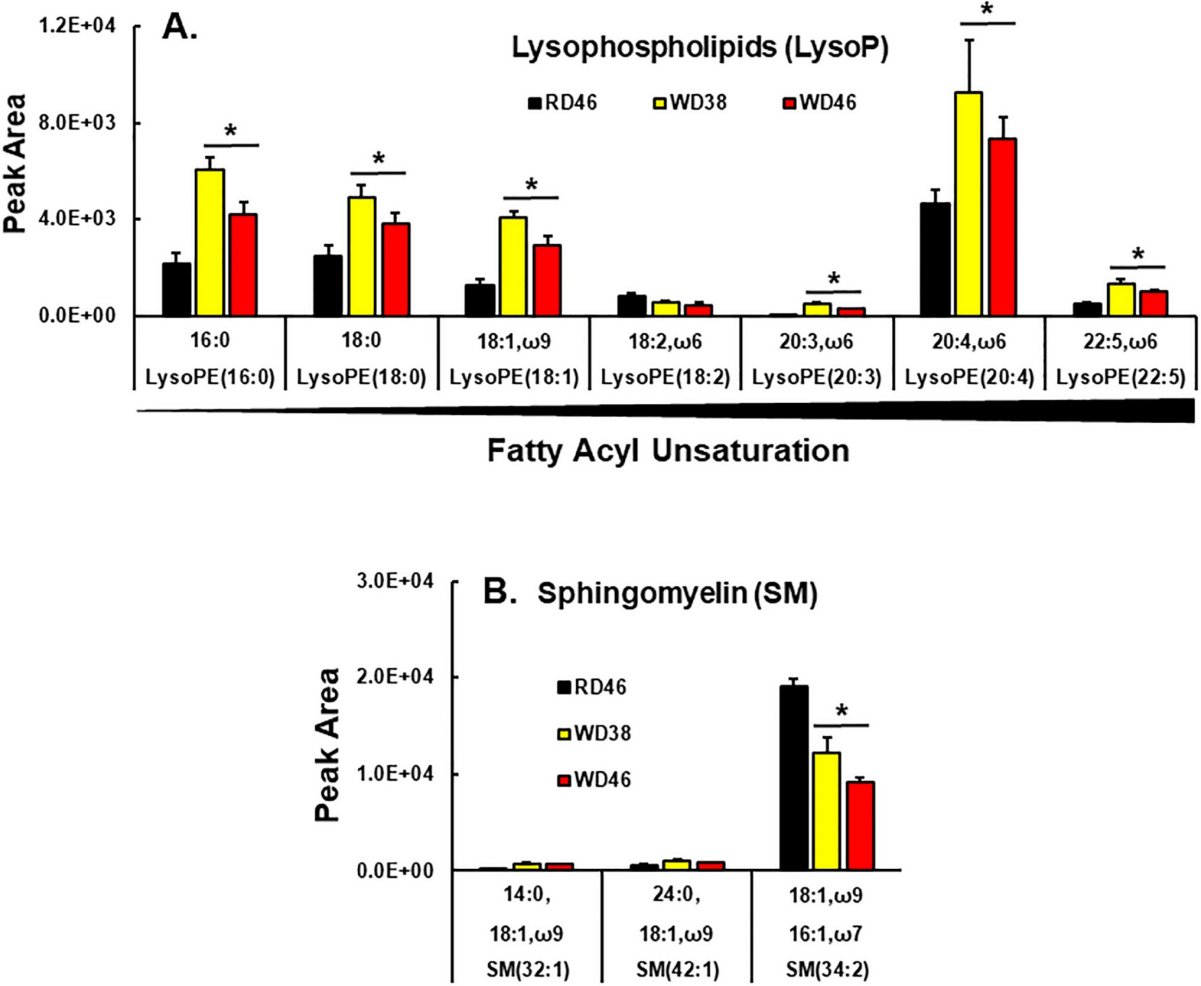
Western diet effects on hepatic lysophospholipids (LysoP) and sphingomyelins (SM). Lysophospholipids (LysoP) (**A**) and sphingomyelins (SM) (**B**) were quantified using an un-targeted LC/MS approach (Materials and methods). Results are presented as peak area, mean ± SEM, N = 7–8 samples; *, FDR <0.05 versus the RD46 group. LysoP are arranged as increasing fatty acyl unsaturation within specific LysoP.

Lysophospholipids (LysoP) are intermediates of phospholipid synthesis (Kennedy pathway) [90] and also arise from phospholipase action during membrane remodeling (Lands Pathway) [91]. Our analysis of LysoP did not distinguish between sn1 vs sn2 acyl chain positions (Fig 12A). Of the seven annotated LysoP, six increased in livers of WD-fed mice. All LysoP increasing in response to the WD contained SFA, MUFA or ω6 PUFA; none contain ω3 PUFA. Taken together, these findings suggest that increases in hepatic SFA and MUFA promotes remodeling of membrane glycerophospholipids acyl chain content resulting in a loss of ω3 PUFA acyl chains from GpCho, GpEtn, GpSer, GpIns, but increases of MUFA and ω3 & ω6 PUFA in GpGro. Such changes in glycerophospholipid PUFA content affects membrane fluidity and signaling from membranes. In addition, the increase of PUFA in cardiolipin precursors may sensitize mitochondria to oxidative damage.

Lastly, we annotated 3 sphingomyelin (**SM**) species (Fig 12B). SM are key plasma membrane lipids, often found in lipid rafts in association with cholesterol [92]. Sm 34:2 was the most abundant Sm detected in liver; and SM 34:2 was ~50% lower in livers of mice in the WD46 group.

To assess potential mechanisms accounting for changes in membrane lipids, we examined hepatic expression of enzymes associated with glycerophospholipid and sphingolipid metabolism (Fig 9A). Two enzymes associated with the Kennedy pathway for *de novo* phospholipid synthesis are also involved in neutral lipid synthesis, e.g., Gly Kin & Gpatm. Only *Gpatm* expression was suppressed in livers of WD-fed mice. In contrast, enzymes involved in membrane remodeling (LpCat 1, 2 & 4) and sphingolipid synthesis (SpTlc 2) were induced ≤2-fold in livers of WD-fed mice. LpCat2 was the most responsive (5-fold induction) to the WD. Expression of enzymes involved in cardiolipin synthesis and turnover (*Crls1*, *Taf*) were attenuated <50%. Expression of SpTlc2 was the only enzyme involved in sphingolipid metabolism affected by the WD-fed mice. Changes in the mRNA abundance encoding these enzymes cannot fully explain the changes in glycerophospholipid and sphingolipid abundance and composition. As such, other mechanisms, such as substrate availability and post-translational mechanisms regulating enzyme activity will need to be examined for their involvement in WD-induced changes in membrane lipid composition.

### Diet effects on oxylipins

**Fig 13:**
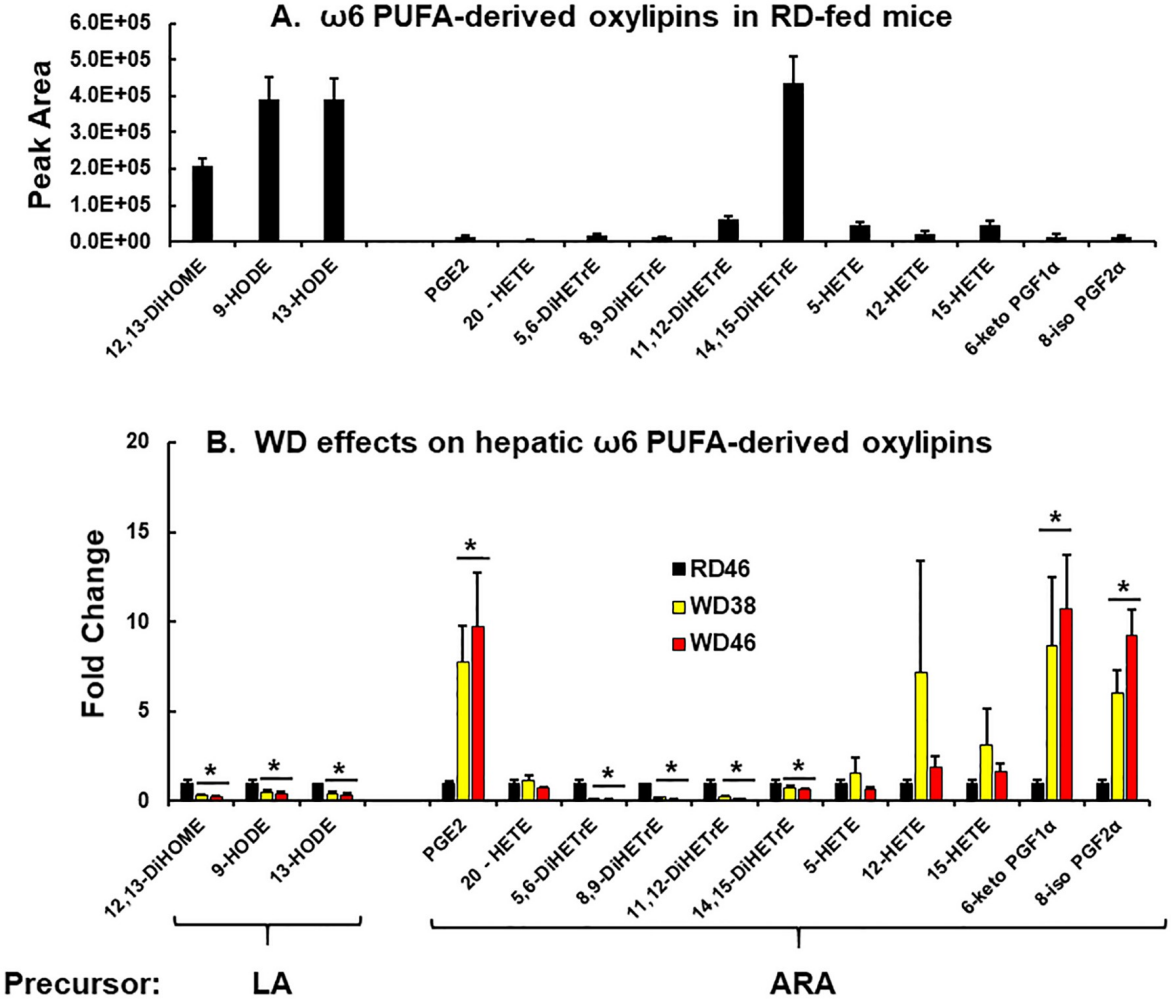
Western diet effects on hepatic ω6-PUFA derived oxylipins. ω6-PUFA derived oxylipins were quantified using a targeted LC/MS approach (Materials and methods). **A**: ω6 PUFA-derived oxylipins in RD-fed mice. **B**: Diet effects on ω6 PUFA-derived oxylipins. Results are presented as fold change in oxylipins induced by the WD. (mean ± SEM, N = 7–8 samples), *, FDR <0.05 versus the RD46 group. DiHOME, dihydroxyoctadecaenoic acid; HODE, hydroxyoctadecaenoic acid; PGE, prostaglandin E; PGF, prostaglandin F; HETE, hydroxyeicosatrienoic acid; DiHETrE, dihydroxyeicosatrienoic acid; LA, linoleic acid; ARA, arachidonic acid.

**Fig 14:**
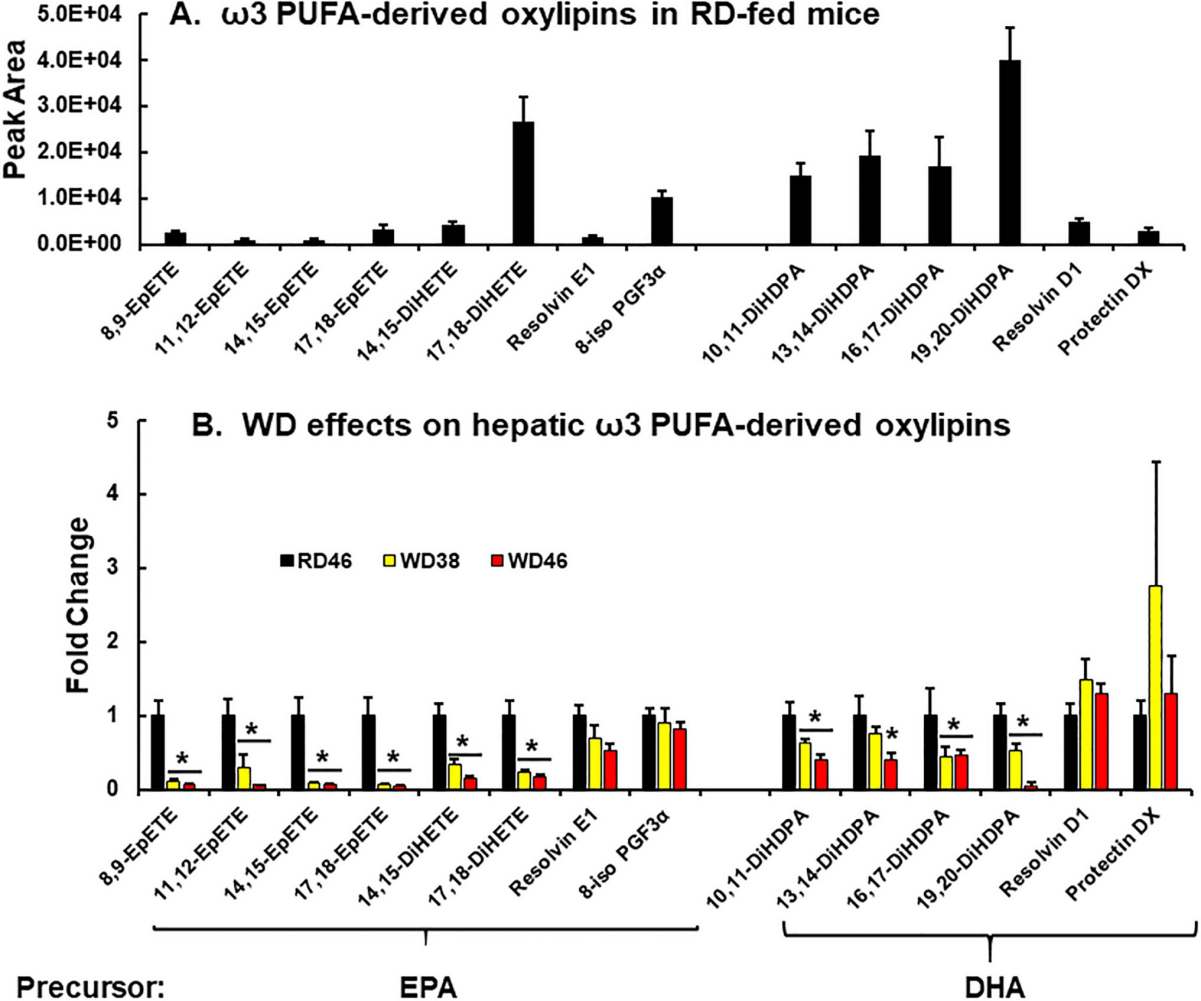
Western diet effects on hepatic ω3-PUFA derived oxylipins. ω3 PUFA derived oxylipins were quantified using a un-targeted LC/MS approach (Materials and methods)s. **A**: ω3 PUFA-derived oxylipins in RD-fed mice. **B**: Diet effects on ω3 PUFA-derived oxylipins. Results are presented as fold change in oxylipins induced by the WD. (mean ± SEM, N = 7–8 samples), *, FDR <0.05 versus the RD46 group. EpETE, epoxyeicosatetraenoic acid; DiHETE, dihydroxyepoxyeicosatetraenoic acid, DiHDPA, dihydroxy docosapentaenoic acid; PGF3α, prostaglandin F3α; EPA, eicosapentaenoic acid; DHA, docosahexaenoic acid.

Oxylipins represent the third class of lipids examined in our analysis (Figs 13 and 14). Oxylipins play key regulatory roles in multiple pathways [93]. Our targeted LC-MS/MS analysis of oxylipins included only the non-esterified fraction of hepatic lipids. Both enzymatic and non-enzymatic pathways generate these oxylipins. Oxylipins annotated in this analysis were derived from ω6 PUFA, i.e., linoleic acid (**LA**) & arachidonic acid (**ARA**) and ω3 PUFA, e.g., eicosapentaenoic acid (**EPA**) and docosahexaenoic acid (**DHA**). A total of hepatic 28 oxylipins were annotated and 20 oxylipins were significantly (*q* < 0.05) affected by the WD. The heat map (S4 Fig) provides an overview of the WD effects on oxylipin content in female *Ldlr* ^*-/-*^ mouse liver.

**ω6 PUFA-derived oxylipins**. LA-derived oxylipins are abundant in liver of the RD46 group and include 12,13-DiHOME (12,13-dihydroxy-9Z-octadecenoic acid), 9-HODE (9-hydroxyoctadecadienoic acid) & 13-HODE (13-hydroxyoctadecadienoic acid) (Fig 13A). Hepatic levels of these LA-derived oxylipins are lower (≤50%) in mice fed the WD for 38 or 46 wks (Fig 13B). These oxylipins are biomarkers of NAFLD progression [94]. For example, patients with stage 1 and stage 2 liver steatosis had significantly lower levels 9-HODE [95]. 9-HODE can be produced enzymatically by 5-lipoxygenase (5-LOX) and non-enzymatically from LA as a result of elevated oxidative stress. The WD increased lipid oxidative stress markers (6-keto PGF1α, 8-iso-PGF2α, 8-iso-PGF3α), but had no significant effect on Lox5 expression (Figs 13 and 14). Changes in hepatic oxidative stress cannot account for the decline in the LA-derived oxylipins. A more likely explanation is the decline in the relative hepatic abundance of ω6 PUFA (Fig 6C and 6D).

In contrast to LA-derived oxylipins, most ARA-derived oxylipins are in low abundance in mouse liver (Fig 13A). Of these, the prostaglandins (PGE2, 6-keto PGF1α and 8-iso PGF2α) are significantly increased (≤ 6-fold) in livers of WD-fed mice (Fig 13B). Cyclooxygenases (*Cox1 & Cox2*) are key enzymes required to initiate prostaglandin synthesis; and PGE2 is a key prostaglandin involved in inflammation. In contrast, 6-keto PGF1α is a non-enzymatic degradation product of prostacyclin (PGI2), while 8-iso PGF2α is an isoprostane (IsoP2), a non-enzymatic oxidation product of ARA. Both are generated in states of oxidative stress by ROS, like O_2_^-^ (superoxide radical), OH (hydroxyl radical or H_2_O_2_ (hydrogen peroxide). The increase in these ARA-derived pro-inflammatory and oxidative stress markers is associated with increases in transcriptomic markers of hepatic inflammation and oxidative stress in WD-fed mice (Fig 4B and 4C).

Leukotrienes are also low abundance oxylipins in mouse liver. Leukotrienes (5-, 12- and 15-HETE) are products of specific lipoxygenases, while 20-HETE is a cytochrome P450 (Cyp450) product. Of these, only 12-HETE increased in livers of WD-fed mice. However, the variance was too high to be significant. The DiHETEs are epoxide hydrolase products of epoxy-fatty acids. Epoxy fatty acids are enzymatic products of ARA catalyzed by *Cyp2C or Cyp5J*. These oxidized PUFA are generally regarded as beneficial in promoting organ and tissue regeneration and attenuating inflammation [96, 97]. Epoxy-fatty acids are converted to dihydroxy fatty acids (DiHETE) by soluble epoxide hydrolase (Ephx2) activity. While Ephx1 is associated with microsomes, Ephx2 is soluble. We detected no ω6-PUFA derived epoxide products of ARA. However, we quantified several DiHETrE, derived from epoxy-derivatives of ARA. Most DiHETrE were lower in livers of WD-fed mice.

**ω3 PUFA-derived oxylipins**. We quantified eight oxylipins derived from eicosapentaenoic acid (EPA, 20:5,ω3) and six oxylipins derived from docosahexaenoic acid (DHA, 22:6,ω3) (Fig 14A). The most abundant ω3 PUFA-derived oxylipins are dihydroxy oxylipins derived from EPA (17,18-DiHETE) and DHA (10,11 DiHDPA, 13,14-DiHDPA, 16,17-DiHDPA, 19,20-DiHDPA). The EPA-derived epoxy lipids (8,9-EpETE, 11,12-EpETE, 14,15-EpETE, 17,18-EpETE) and the anti-inflammatory resolvins (Rev E1 & Rev D1) and protectin DX are in low abundance in mouse liver. The non-enzymatic oxidation product of EPA, i.e., 8-iso PGF3α (IsoP3) was also measured.

All of the EPA- and DHA-derived epoxy and dihydroxy oxylipins were significantly lower (≤ 50%) in livers from the WD-fed mice. There was no significant diet effect on EPA or DHA-derived selective pro-resolving mediators [resolvins (Rev E1, Rev D1), protectin DX] or the EPA-derived isoprostane, 8-Iso-PGF3α.

**Fig 15:**
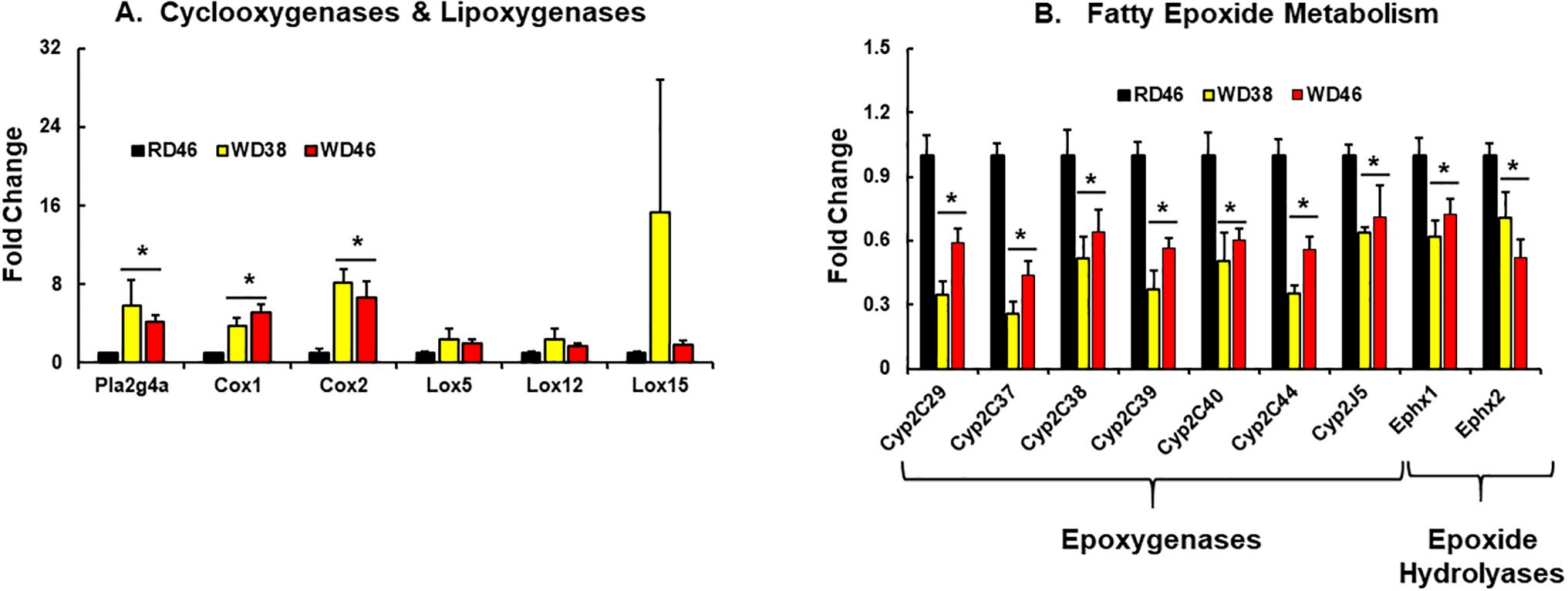
**Western diet effects on hepatic expression of enzymes linked to oxylipin metabolism:** Cyclooxygenases and lipoxygenases (A) and enzymes involved in fatty epoxide metabolism (B). Transcript abundance was quantified as described above. Results are expressed as mRNA abundance, as fold change; mean ± SEM, N = 7–8 samples, *, FDR <0.05 versus the RD46 group. [**A**: PLA2g4a, phospholipase A2g4a; Cox, cyclooxygenase; Lox, lipoxygenase]; [**B**: Cyp2C, cytochrome P450 2C class; Cyp2J, cytochrome P450 2J class; Ephx1, epoxide hydrolase (microsomal); Ephx2, epoxide hydrolase (soluble)].

To gain insight into factors controlling oxylipins levels, we quantified the expression of enzymes involved in oxylipin synthesis, including phospholipase A2 (Pla2g4a), cyclooxygenases (*Cox 1 & 2*), lipoxygenases (Lox 5, 12 and 15), epoxygenase subtypes Cyp2C (*Cyp2C29*, *Cyp2C27*, *Cyp2C38*, *Cyp2C39*, *Cyp2C40*, *Cyp2C44* and *Cyp2J5*), as well as the two epoxy hydrolases (*Ephx1* [microsomal] & *Ephx 2* [soluble]) (Fig 15A and 15B).

Phospholipase A2 (Pla2g4a) is a key enzyme that hydrolyzes fatty acyls from the sn2 position of membrane phospholipids. The product of this reaction serves as substrate for COX, LOX, Cyp2C and Cyp2J. The expression of *Pla2g4a* and cyclooxygenases (*Cox 1 & 2)* were significantly higher (≤5-fold) in livers of WD fed mice. While the increase in PGE2 is likely due to increased Cox1 & 2 expression, increased 6-keto-PGF1α, 8-iso-PGF2α abundance is associated with increased oxidative stress in the liver (Figs 4C, 13 and 15A). WD did not significantly increase lipoxygenase expression (*Lox 5*, *12*, *& 15)*. Expression all seven Cyp2 subtypes (*Cyp2C29*, *Cyp2C37*, *Cyp2C38*, *Cyp2C39*, *Cyp2C40*, *Cyp2C44*, *Cyp2J5*), however, was attenuated (≤50%) in livers of WD-fed mice. Decreased epoxy- and dihydroxy products of ARA, EPA and DHA are likely due to reduced enzyme expression, reduced membrane associated ARA, EPA and DHA and overall lower levels of hepatic C_18-22_ ω3 PUFA (Figs 6C, 10, 11, 14 and 15A).

### Associations between major NASH markers and specific hepatic features

**Fig 16:**
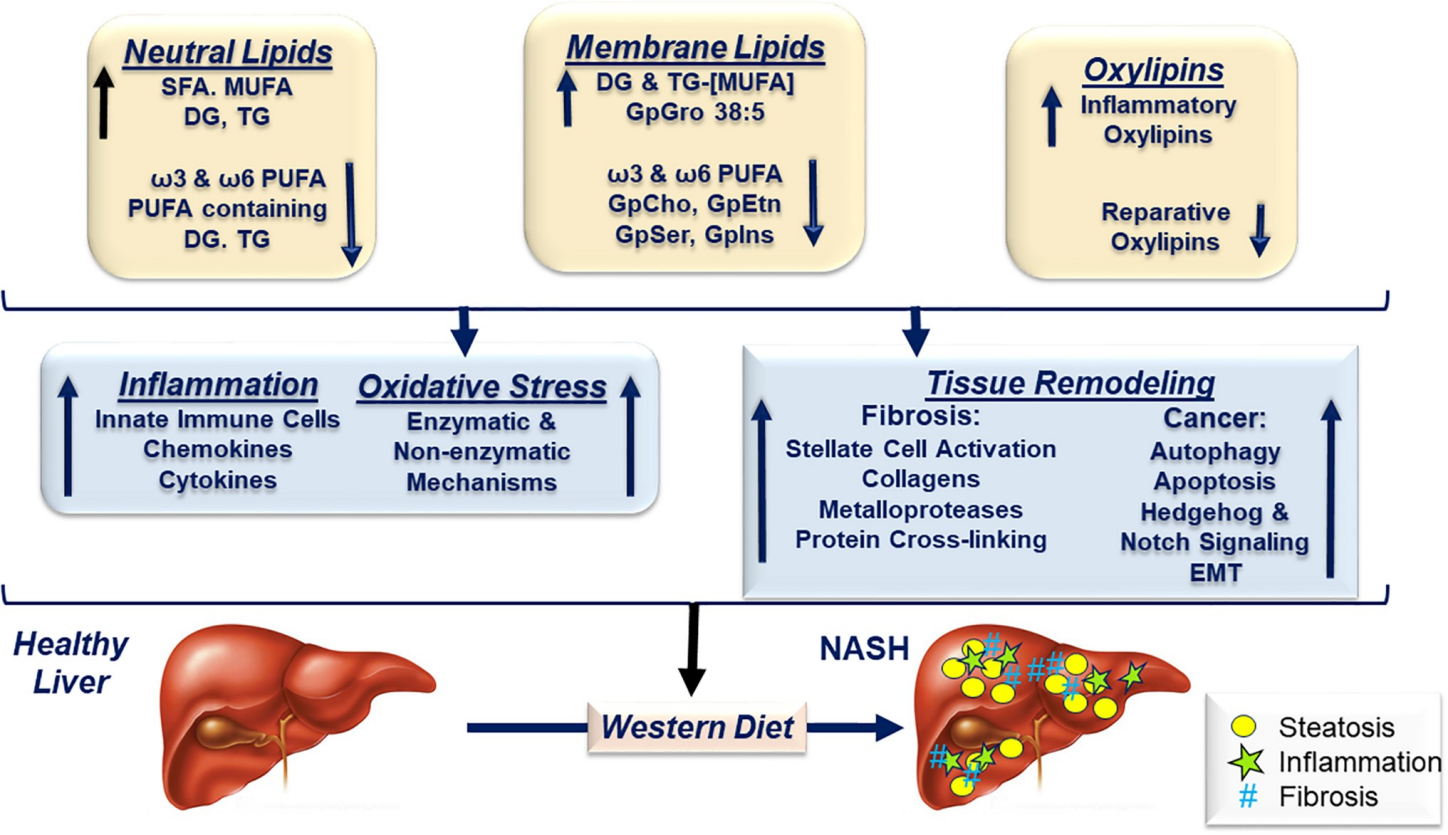
Summary of western diet effects on hepatic lipids and gene expression in female *Ldlr* ^*-/-*^ mice.

Finally, we took advantage of the heterogeneity in response to the WD reported in Fig 3 to carry out an unbiased statistical approach to identify associations between key NASH markers and transcriptomic and lipidomic features. Accordingly, “Pattern Hunter” in Metabolanalyst was used to identify associations (Tables 3–6 and Fig 16) [38]. NASH markers used in this analysis included steatosis (total hepatic fatty acyls), inflammation (*TNFα*), oxidative stress (*Ncf2*), fibrosis (*Col1A1*), notch signaling (*Hey1*), EMT (*S1004A*), cancer (Gpc3), ω6 PUFA-derived oxylipins (PGE2) and ω3 PUFA-derived oxylipins (19,20-DiHDPA). Results in Tables 3–6 include the top 10 features that were positively and negatively associated with each NASH marker. The features are rank ordered based on the correlation coefficient (CC) and statistical significance (FDR value).

**Table 3:** Top 10 lipid and gene expression markers associated with hepatosteatosis and inflammation^1^.

Pathway		Steatosis			Inflammation	
Marker		*Hepatic fatty acyls*			*TNFα*	
Association	Feature	CC^2^	FDR^3^	Feature	CC	FDR
Positive	SFA	0.99	1.3E-25	Mcp1	0.98	2.8E-13
Positive	16:0	0.99	1.1E-20	LpCat2	0.96	4.8E-11
Positive	16:1,ω7	0.99	3.4E-18	MIS^4^	0.95	4.7E-10
Positive	MUFA	0.99	3.5E-18	GpGro 38:5	0.94	6.9E-10
Positive	18:1,ω9	0.99	7.6E-18	Ncf2	0.94	2.5E-09
Positive	14:0	0.99	5.9E-17	TG 54:3	0.93	9.2E-09
Positive	DG 34:1	0.96	7.3E-12	DG 34:1	0.92	1.0E-08
Positive	TG 50:2	0.95	7.4E-11	DG 36:1	0.92	1.3E-08
Positive	TG 58:3	0.95	1.8E-10	18:1,ω9	0.92	1.5E-08
Positive	DG 32:1	0.95	1.9E-10	MAS^5^	0.92	1.8E-08
Negative	ω6 Index	-0.96	1.5E-11	ω6 Index	-0.95	5.1E-10
Negative	ω3 Index	-0.96	2.5E-11	ω3 Index	-0.94	2.5E-09
Negative	GpCho 38:4	-0.93	3.0E-09	GpEtn 36:2	-0.92	1.8E-08
Negative	GpEtn 38:6	-0.89	1.3E-07	GpCho 36:5	-0.92	2.0E-08
Negative	GpSer 40:6	-0.89	1.4E-07	GpEtn 38:6	-0.91	2.5E-08
Negative	TG 58:9	-0.88	2.0E-07	GpCho 36:2	-0.90	6.2E-08
Negative	GpCho 36:5	-0.87	6.3E-07	GpEtn 34:2	-0.90	6.5E-08
Negative	Total GpSer	-0.86	1.3E-06	GpEtn 36:5	-0.89	8.1E-08
Negative	GpEtn 36:2	-0.84	2.6E-06	GpSer 40:6	-0.89	8.1E-08
Negative	Fdps	-0.84	2.8E-06	GpCho 38:4	-0.87	2.8E-07

^1^Associations between NASH features were determined using the statistical package "Pattern Hunter" in Metabolanalyst [37, 38, 53]. The markers chosen for this analysis were based on the most robust response to the WD within each pathway.

^2^Correlation coefficient;

^3^False discovery rate;

^4^MIS, microsteatosis;

^5^MAS, macrosteatosis

**Table 4:** Top 10 lipid and gene expression markers associated with oxidative stress and fibrosis^1^.

Pathway		Oxidative Stress			Fibrosis	
Marker		*Ncf2*			*Col1A1*	
Association	Feature	CC^2^	FDR^3^	Feature	CC	FDR
Positive	LpCat2	0.98	4.4E-13	Mcp1	0.90	4.0E-07
Positive	SpTlc2	0.95	4.1E-10	Liver Wt	0.90	4.0E-07
Positive	MIS^4^	0.94	2.1E-09	TNFα	0.88	1.6E-06
Positive	TNFα	0.94	3.7E-09	Change BW	0.88	1.6E-06
Positive	LpCat1	0.93	9.6E-09	AST U/L	0.88	2.4E-06
Positive	GpGro 38:5	0.92	2.4E-08	TG 54:3	0.87	2.7E-06
Positive	DG 36:1	0.91	3.8E-08	MAS^5^	0.87	2.7E-06
Positive	GpEtn 40:5	0.91	5.5E-08	GpGro 38:5	0.87	2.7E-06
Positive	18:1,ω9	0.90	1.0E-07	LpCat2	0.87	2.7E-06
Positive	SM 32:1	0.90	1.0E-07	Total GpGro	0.85	6.1E-06
Negative	GpCho 36:5	-0.93	1.2E-08	GpEtn 36:2	-0.85	7.0E-06
Negative	GpEtn 38:6	-0.92	2.7E-08	GpEtn 34:2	-0.85	8.2E-06
Negative	GpEtn 36:5	-0.92	2.7E-08	GpEtn 36:5	-0.83	1.3E-05
Negative	ω6 Index	-0.91	4.3E-08	ω3 Index	-0.83	1.3E-05
Negative	TG 58:9	-0.91	4.3E-08	ω6 Index	-0.83	1.3E-05
Negative	ω3 index	-0.91	5.1E-08	GpCho 36:2	-0.82	2.3E-05
Negative	Pdk4	-0.89	1.2E-07	GpEtn 38:6	-0.81	3.1E-05
Negative	11,12-EpETE	-0.88	2.3E-07	GpSer 40:6	-0.79	5.4E-05
Negative	GpCho 38:4	-0.88	2.3E-07	GpEtn 34:3	-0.79	6.1E-05
Negative	GpEtn 34:2	-0.88	2.6E-07	GpCho 36:5	-0.78	8.3E-05

^1^Associations between NASH features were determined using the statistical package "Pattern Hunter" in Metabolanalyst [37, 38, 53]. The markers chosen for this analysis were based on the most robust response to the WD within each pathway.

^2^Correlation coefficient;

^3^False discovery rate;

^4^MIS, microsteatosis;

^5^MAS, macrosteatosis

**Table 5:** Top 10 lipid and gene expression markers associated with notch signaling (Notch), epithelial mesenchymal transition (EMT) and cancer^1^.

Pathway		Notch			EMT			Cancer	
Marker		*Hey1*			*S100A4*			*Gpc3*	
Association	Feature	CC^2^	FDR^3^	Feature	CC	FDR	Feature	CC	FDR
Positive	CtsS	0.97	6.4E-13	Mpk	0.96	1.1E-10	CtsS	0.83	9.1E-05
Positive	HeyL	0.97	2.1E-12	MIS	0.95	5.5E-10	HeyL	0.82	9.1E-05
Positive	CD68	0.96	2.3E-11	TG 51:1	0.92	2.3E-08	Sox4	0.81	9.1E-05
Positive	Tgfβ1	0.95	4.2E-10	DG 34:1	0.92	2.4E-08	CD40	0.81	9.1E-05
Positive	CD44	0.95	4.8E-10	18:1,ω9	0.92	2.4E-08	Hey1	0.81	9.1E-05
Positive	MIS^4^	0.95	5.1E-10	Hey1	0.91	2.8E-08	Cox2	0.81	9.1E-05
Positive	Bcl2	0.94	2.3E-09	MUFA	0.91	2.8E-08	Hmox1	0.81	9.1E-05
Positive	Casp1	0.93	3.8E-09	14:0	0.91	2.8E-08	CD68	0.80	9.1E-05
Positive	F4/80	0.92	1.0E-08	HeyL	0.91	3.3E-08	Tgfβ1	0.80	1.1E-04
Positive	Cxcl14	0.92	1.0E-08	DG 36:1	0.91	4.5E-08	Cxcl14	0.79	1.1E-04
Negative	GpCho 36:5	-0.92	1.1E-08	ω3 index	-0.94	9.7E-10	8,9-EpETE	-0.82	9.1E-05
Negative	ω6 Index	-0.92	1.1E-08	ω6 Index	-0.94	9.7E-10	GpEtn 42:8	-0.81	9.1E-05
Negative	ω3 index	-0.92	1.1E-08	GpCho 36:5	-0.92	2.0E-08	GpIns 36:2	-0.81	9.1E-05
Negative	GpSer 40:6	-0.90	5.9E-08	TG 58:9	-0.92	2.4E-08	GpCho 36:4	-0.80	9.1E-05
Negative	TG 58:9	-0.90	6.8E-08	GpSer 40:6	-0.91	3.3E-08	TG 62:13	-0.80	1.1E-04
Negative	Total GpSer	-0.90	8.0E-08	Total GpSer	-0.90	6.3E-08	Tgh	-0.79	1.1E-04
Negative	Pdk4	-0.89	9.4E-08	GpCho 38:4	-0.89	1.4E-07	TG 58:9	-0.79	1.1E-04
Negative	11,12-EpETE	-0.89	1.2E-07	GpEtn 38:6	-0.88	2.1E-07	GpIns 40:6	-0.79	1.1E-04
Negative	GpEtn 36:5	-0.88	2.3E-07	GpEtn 36:2	-0.88	2.7E-07	GpCho 36:5	-0.78	1.5E-04
Negative	GpEtn 36:2	-0.88	2.8E-07	11,12-EpETE	-0.88	2.9E-07	GpCho 40:8	-0.78	1.5E-04

^1^Associations between NASH features were determined using the statistical package "Pattern Hunter" in Metabolanalyst [37, 38, 53]. The markers chosen for this analysis were based on the most robust response to the WD within each pathway.

^2^Correlation coefficient;

^3^False discovery rate;

^4^MIS, microsteatosis

**Table 6:** Top 10 lipid and gene expression markers associated with ω6 PUFA- and ω3 PUFA-derived oxylipins^1^.

Pathway		ω6 Oxylipins			ω3 Oxylipins	
Marker		PGE2			19,20-DiHDPA	
Association	Feature	CC^2^	FDR^3^	Feature	CC	FDR
Positive	8-iso PGF2α	0.92	7.0E-08	GpEtn 40:8	0.73	6.3E-03
Positive	LpCat2	0.91	2.4E-07	DG 40:8	0.71	6.3E-03
Positive	Ncf2	0.89	1.4E-06	Total ω3 oxylipins	0.71	6.3E-03
Positive	6-keto PGF1α	0.88	2.3E-06	TG 62:13	0.71	6.3E-03
Positive	SpTlc2	0.87	2.8E-06	GpSer 40:6	0.67	1.1E-02
Positive	TNFα	0.86	5.5E-06	TG 58:8	0.66	1.1E-02
Positive	LpCat4	0.85	9.2E-06	GpCho 36:5	0.66	1.1E-02
Positive	LW%BW	0.84	1.2E-05	GpEtn 36:5	0.66	1.1E-02
Positive	MIS^4^	0.84	1.5E-05	GpEtn 34:3	0.66	1.1E-02
Positive	Liver Wt	0.82	2.4E-05	TG 52:4	0.66	1.1E-02
Negative	GpCho 36:5	-0.87	4.6E-06	LpCat4	-0.75	5.1E-03
Negative	GpEtn 36:5	-0.85	1.1E-05	Gadd45	-0.71	6.3E-03
Negative	TG 62:13	-0.84	1.2E-05	PGE2	-0.71	6.3E-03
Negative	GpEtn 38:6	-0.84	1.2E-05	Ncf2	-0.67	1.1E-02
Negative	TG 58:9	-0.84	1.3E-05	LpCat2	-0.66	1.1E-02
Negative	GpEtn 40:6	-0.83	2.4E-05	8-iso PGF2α	-0.66	1.1E-02
Negative	GpEtn 34:2	-0.82	3.4E-05	SpTlc2	-0.66	1.1E-02
Negative	GpEtn 34:3	-0.81	3.4E-05	Hes1	-0.66	1.1E-02
Negative	Total GpSer	-0.81	3.4E-05	Ccl22	-0.63	1.4E-02
Negative	GpEtn 36:2	-0.81	3.4E-05	Cox1	-0.63	1.4E-02

^1^Associations between NASH features were determined using the statistical package "Pattern Hunter" in Metabolanalyst [37, 38, 53]. The markers chosen for this analysis were based on the most robust response to the WD within each pathway.

^2^Correlation coefficient;

^3^False discovery rate;

^4^MIS, microsteatosis.

**Steatosis**. Features positively associated with steatosis included SFA, MUFA and MUFA-containing DG & TG (Table 3 and Fig 16). Features negatively associated with hepatic steatosis include the relative abundance of PUFA (ω3 and ω6 index) and PUFA-containing TG, GpCho, GpEtn, GpSer & *Fdps*. The decline in membrane PUFA gives the appearance of essential fatty acid deficiency. However, the WD is an essential fatty acid sufficient diet [29]. Markers of essential fatty acid deficiency include increased DNL and mead acid (20:3,ω9) synthesis [98]. While hepatic fatty acid (GC) analyses did not detect mead acid or increased expression of enzymes involved in DNL (Figs 6C and 7B), untargeted LC-MS/MS analysis detected glycerophospholipids containing 20:3,ω9, i.e., GpCho 40:5; GpEtn 38:3; GpIns 36:3 & 38:3 and GpGro 38:4 (Figs 10 and 11). Formation of 20:3,ω9 is likely due to the high abundance of 18:1,ω9 in the livers of WD-fed mice (Fig 5) and off-target actions of Fads1 & Fads2 [72].

Other mechanisms contributing to hepatosteatosis include suppression of FAO, increased fatty acid uptake, neutral lipid synthesis and attenuated VLDL secretion. The WD did not affect hepatic expression of PPARα or its target genes involved in FAO (Cpt1A & HMG CoA Syn2), fatty acid binding protein (Fabp1) (Fig 7C) or increased neutral lipid synthesis (Fig 9A). Instead, the expression of two enzymes involved in TG catabolism, i.e., *Ces1g* and *Tgh*, was suppressed ≤50% by the WD (Fig 9B). Ablation of Tgh [82] or Ces1g [83] affects hepatic neutral lipid content. We also examined the expression of a third enzyme involved in TG catabolism, i.e., ATGL (Fig 9B). Others have reported that mice fed high fat diets display low levels of ATGL expression in adipose tissue [99]. This effect was linked to high fat diet-induced Snail1; and Snail suppressed ATGL expression [99]. While the WD induced Snail1 expression in *Ldlr* ^*-/-*^ mouse liver (Fig 5C), there was no significant effect of the WD on hepatic ATGL expression (Fig 9B). As such, WD-mediated suppression of Tgh and Ces1g may be one explanation for increased neutral lipid content in livers of WD-fed *Ldlr* ^*-/-*^ mice. As noted above, TG catabolism is required for assembly and lipidation of ApoB for VLDL assembly and secretion [84, 85]. Decreased TG catabolism, coupled with decreased VLDL secretion may account for the increased in hepatic neutral lipid in WD-fed mice.

Diacylglycerols and triacylglycerols were enriched in MUFA in livers of mice fed the WD (Fig 8). The increase in hepatic MUFA, can be explained, at least in part, by increased Scd1 expression (Fig 7), as well as the high dietary content of SFA & MUFA [29]. Scd1 expression is regulated by multiple transcription factors, including, SREBP1, ChREBP, PPARα, PPARγ2 and LXR [73, 74]. The lack of diet effects on Cpt1a or HMG CoA Syn2, two PPARα target genes, argues against PPARα activation in the control of Scd1. Moreover, previous studies indicated that hepatic ChREBP nuclear abundance was unaffected in WD-fed *Ldlr*^*-/-*^ mice [28]. Herein, we report that SREBP1c and PPARγ2 were induced in livers of WD-fed mice (Fig 7A). These results are consistent with reports of others [74, 100]; PPARγ2 expression is associated with increased hepatic neutral lipid storage.

While we did not measure LXR (α or β) directly, we present evidence (5S Fig) suggesting that LXR was activated by the WD. Specifically, the LXR target gene, Cyp7α, was induced ≤ 1.5-fold in livers of mice fed the WD for 38 & 46 wks. LXR is activated by oxysterols [101] and the WD contains moderately high (0.15% w/w) cholesterol, which likely increases hepatic oxysterol formation. As such, the massive increase in hepatic SFA and MUFA and diacylglycerols & triacylglycerols is likely due to: a) high SFA and MUFA content of the WD; b) induction/activation of transcription factors (SREBP1c, PPARγ2, LXR) controlling lipid synthesis; c) modulation in TG catabolism through (Tgh and Ces3) and 4) possibly impaired VLDL secretion.

**Inflammation, oxidative stress and fibrosis**. Expression levels of *TNFα*, *Ncf2* and *Col1A1* were used as markers for inflammation, oxidative stress and fibrosis, respectively (Tables 3 and 4 and Fig 16). The correlation analyses revealed that these pathways share many common features. The common features positively associated with these markers include enzymes involved in membrane remodeling (LpCat1 & LpCat2), a chemokine (Mcp1), neutral lipids containing SFA and MUFA, and a phosphatidylglycerol containing 18:1,ω9 and 20:4,ω6 (GpGro 38:5). Phosphatidylglycerols are cardiolipin precursors; and cardiolipins are complex phospholipids found in the mitochondrial matrix. The correlation coefficients for these associations are high (r > 0.84).

Common features that were negatively associated with inflammation, oxidative stress and fibrosis include the ω3 and ω6 index and several PUFA-containing glycerophospholipids (GpCho, GpEtn, GpSer). While a negative association between PUFA, particularly ω3 PUFA, and inflammation has been reported previously [102, 103], these new findings identify specific PUFA-containing membrane lipids affected by the WD.

Particularly relevant is the major change in fatty acid type in membrane lipids. Inflammation, oxidative stress and fibrosis are positively associated with membrane lipids enriched in SFA and MUFA and negatively associated with phospholipids containing PUFA. This is particularly evident in GpEtn and GpSer, which are typically associated with the inner plasma membrane and intracellular membranes, e.g., ER, golgi, etc. This change in membrane lipid composition can have broad effects on hepatic physiology resulting from changes in trafficking and membrane-associated cell signaling.

**Notch signaling, EMT and hepatic cancer**. Notch signaling is a highly conserved morphogenic signaling mechanisms that control cell fate decisions, morphogenesis, proliferation and apoptosis during development, EMT and tissue repair [62, 104]. We combined the analysis of Notch signaling with EMT & hepatic cancer markers since these pathways are upregulated in hepatic malignancies, such as primary hepatocellular cancer (HCC) and intrahepatic cholangiocarcinoma [62, 68–70, 105]. Accordingly, Hey1, S100A4 and Gpc3 were used as markers for notch signaling, EMT and hepatic cancer, respectively (Table 5 and Fig 16).

Hey1 and HeyL, notch-associated transcription factors, were positively associated with both EMT and cancer. Three lipids (GpCho 36:5, TG 58:9 & 11,12-EpETE and 8,9-EpETE) were negatively associated with the notch, EMT and cancer (Table 5 and Fig 16). These lipids are either complex lipids containing PUFA or oxidized metabolites derived from PUFA. An emerging theme from this analysis is that hepatic PUFA-containing phosphoglyceroholipids, e.g., GpCho, GpEtn, GpSer, are negatively associated with an increased expression of hepatic markers associated with steatosis, inflammation, oxidative stress, fibrosis (Tables 3 and 4), Notch signaling, EMT and cancer (Table 5). Whether these changes in membrane lipids are causally linked to WD-induced hepatic pathology remains to be established.

**ω6 PUFA-derived oxylipins**. Features positively and negatively associated with hepatic PGE2, a cyclooxygenase product, are presented in Table 6. Elevated hepatic PGE2 is positively associated with oxidative stress markers (Ncf2, 8-isoPGF2α, 6-ketoPGF1α), membrane remodeling (LpCat2 & 4), inflammation (*TNFα*), liver weight & liver weight as a % of body weight. Elevated hepatic PGE2 is negatively associated with glycerophospholipids (GpCho, GpEtn, GpSer) and TG containing PUFA. Neither *Cox1* nor *Cox2* are expressed in hepatocytes, but these enzymes are expressed in other liver cells, like macrophage (Kupffer cells) and leukocytes infiltrating liver in response to the WD [106] (Fig 3). Products of these enzymes serve as ligands that bind G-protein receptors and typically play a role in inflammation. However, PGE2 has been reported to inhibit expression of lipogenic enzymes in isolated rat primary hepatocytes through EP3 receptors [106]. Thus, the suppression of Fasn (Fig 7B) may be due to elevated hepatic PGE2.

**ω3 PUFA-derived oxylipins**. The DHA-derived oxylipin, 19, 20-DiHDPA, was the major ω3 PUFA-derived oxylipin detected in liver (Fig 14). This dihydroxy oxylipin is derived from epoxygenase-generated 19, 20 EpDPA. Factors positively associated with 19, 20-DiHDPA include multiple glycerophospholipids (GpEtn, GpSer, GpCho) and DG & TG containing PUFA. Negatively associated features include expression markers of membrane remodeling (*LpCat 2 & 4*), sphingolipid synthesis (*SpTlc2*), oxidative stress (*Ncf2*), inflammation (*Ccl22*); prostaglandin synthesis (PGE2, *Cox1*), a notch regulated transcription factor (*Hes1*), and *Gadd45*, a transcription factors associated with DNA repair and cellular stress.

Epoxide products of PUFA are generally regarded as beneficial and play a role in reparative events [96]. Fatty epoxides, however, are rapidly degraded to dihydroxy products by epoxide hydrolases, e.g., Ephx2 (soluble) [96, 97, 107, 108]. Expression of hepatic enzymes involved in the formation of epoxides and dihydroxy fatty acids was suppressed by ~50% in livers of mice consuming the WD (Fig 15). Hepatic levels of 19, 20-DiHDPA, which are dependent on Cyp2C production of the epoxide precursors, are associated with PUFA-enriched membrane lipids and negatively associated with hepatic inflammation, oxidative stress and repair.

## Limitations and conclusions

This report provides new information on the capacity of the WD to promote NASH in a female preclinical mouse model. The WD promotes obesity & dyslipidemia and all of the major hallmarks of NASH, including hepatosteatosis, inflammation, oxidative stress and fibrosis (Table 1 and Figs 3, 4 and 6). Others have reported that NASH is associated with increases in hepatic abundance of transcripts involved in tissue remodeling, namely apoptosis & autophagy [2, 60, 61], hedgehog & notch signaling [62, 63] and cancer [65, 66]. Female *Ldlr* ^*-/-*^ fed the WD have increased hepatic abundance of transcripts encoding proteins involved in these same pathways (Figs 5 and 16).

A key feature accompanying tissue remodeling is the change in the type and abundance of tissue lipid. Our studies provide compelling evidence for WD-induced changes in the acyl chain composition of neutral and polar lipids, particularly hepatic membrane lipids. While this analysis is largely a descriptive, it provides insight into possible mechanisms contributing to the onset and progression of NASH. Chief amongst these is the major shift from PUFA-enriched membrane lipids to SFA & MUFA enriched membrane lipids. Likely mechanisms for this change in membrane composition include the induction of membrane remodeling, i.e., the Land’s pathway, through induction of enzymes like LpCat 1, 2 & 4 (Figs 9–12) and the high substrate availability of SFA & MUFA arising from the diet and disruption of hepatic lipid metabolism. These changes in hepatic lipids have the capacity to affect several regulatory pathways. For example, the increase in hepatic DG containing SFA and MUFA may serve as regulatory ligands controlling protein kinase C subtypes, e.g., PKCε, and tissue insulin sensitivity [109]. The decrease in membrane glycerophospholipids containing PUFA will have effects on membrane fluidity and will affect membrane-associated signaling mechanisms [110] (Fig 16). Compounding these dramatic changes in membrane lipids is the change in the type and abundance of PUFA-derived regulatory oxylipins appearing in the NASH liver. Clearly, the increase in hepatic PGE2 and the ARA-derived oxidative stress marker (8-iso-PGF2α) exacerbates disease progression through induction of inflammatory mechanisms. These changes in hepatic lipid composition are likely major contributors to the onset & progression of WD-induced NASH since supplementing the WD with DHA (at 2% total calories) both restores hepatic C_20-22_ ω3 PUFA content and attenuates disease severity in prevention and remission studies [29, 31, 103].

In conclusion, the female *Ldlr* ^*-/-*^ mouse is a suitable preclinical model to examine NAFLD, from benign steatosis to early stage HCC. Our studies go on to establish that the major markers of NASH are strongly associated with major changes in hepatic neutral and polar lipid acyl chain composition, as well as ω3 and ω6 PUFA-derived oxylipins. The outcome of these studies sets the stage for future mechanistic studies.

## Supporting information

S1 Fig Hepatosteatosis in liver of a WD-fed female *Ldlr* ^*-/-*^ mouse. Liver of a WD-fed mouse (WD38) was fixed in buffered formalin, sectioned and stained with hematoxylin and eosin and photographed at 400x. Regions representing microsteatosis, macrosteatosis and inflammation, i.e., granulocytes, are marked.
(TIF)

S2 Fig Hepatosteatosis and fibrosis in RD- and WD-fed female *Ldlr* ^*-/-*^ mice. Livers of a control and a WD-fed mouse (WD38) were fixed in buffered formalin, sectioned and stained with hematoxylin and eosin and photographed at 200x. Liver from the control group (RD46) shows no signs of hepatosteatosis (H & E) or fibrosis (Sirius Red). Liver from the western diet group (WD38) shows extensive hepatosteatosis (H & E) and fibrosis (Sirius Red).
(TIF)

S3 Fig Principal component analysis (PCA) of lipid changes in the RD46 versus WD38 and WD46 groups. Data included in this analysis included only lipids quantified by LC/MS that were significantly affected by the WD. Liver lipidomic profiles differ depending on the diet and the duration of the diet. PCA was conducted after log transformation on all statistically significant lipids (*q* < 0.05) annotated in liver samples from female mice (positive and negative ion modes merged) and with a coefficient of variation < 30%. Each symbol represents an animal. N = 7–8 per group.
(TIF)

S4 Fig Heat map comparing the oxylipin composition in female *Ldlr*^*-/-*^ WD fed mice after 38 and 46 weeks with the RD group. For each group the average value from 7–8 biological replicates was used. A logarithm data transformation and auto scaling (mean-centered divided by the standard deviation of each variable) were performed in MetaboAnalyst 4.0.in order to produce this visualization map. The represented *q*-values, accounting for differences between the treatments, were obtained as explain in the Materials and Methods section by using GraphPad Prism v7.03.
(TIF)

S5 Fig Western diet effects on hepatic expression of enzymes linked to cholesterol metabolism. Transcript abundance was quantified as described in the Materials and Methods section. Results are expressed as mRNA abundance, Fold Change; mean ± SEM, N = 7–8 samples, *, FDR <0.05 versus the RD46 group. **Hmg CoA Red**: HMG CoA reductase; **Hmg CoA Syn 1**: Hmg CoA synthase 1; **Fdps**: Farnesyl diphosphate synthase; **Cyp7α**: Cytochrome P450 7α.
(TIF)

S1 Table Primer pairs for qRTPCR. (XLSX)

S2 Table Oxylipin standards used for LC/MS analysis. (XLSX)
