# Correction: Lipidomic and transcriptomic analysis of western diet-induced nonalcoholic steatohepatitis (NASH) in female *Ldlr ^-/-^* mice

**DOI:** 10.1371/journal.pone.0216535

**Published:** 2019-05-01

**Authors:** The PLOS ONE Staff

The heading RD38 for the second column of S2 Fig is incorrect. It should read: WD38. Please view the correct S2 Fig below. The publisher apologizes for the error.

## Supporting information

S2 Fig Hepatosteatosis and fibrosis in RD- and WD-fed female *Ldlr ^-/-^* mice. Livers of a control and a WD-fed mouse (WD38) were fixed in buffered formalin, sectioned and stained with hematoxylin and eosin and photographed at 200x. Liver from the control group (RD46) shows no signs of hepatosteatosis (H & E) or fibrosis (Sirius Red). Liver from the western diet group (WD38) shows extensive hepatosteatosis (H & E) and fibrosis (Sirius Red).
(TIF)
