# Supplementary figures and images for: Lipidomic and transcriptomic analysis of western diet-induced nonalcoholic steatohepatitis (NASH) in female Ldlr -/- mice
Source: PLoS One. 2019 Apr 3;14(4):e0214387. doi: 10.1371/journal.pone.0214387 (PMC6447358; doi:10.1371/journal.pone.0214387)

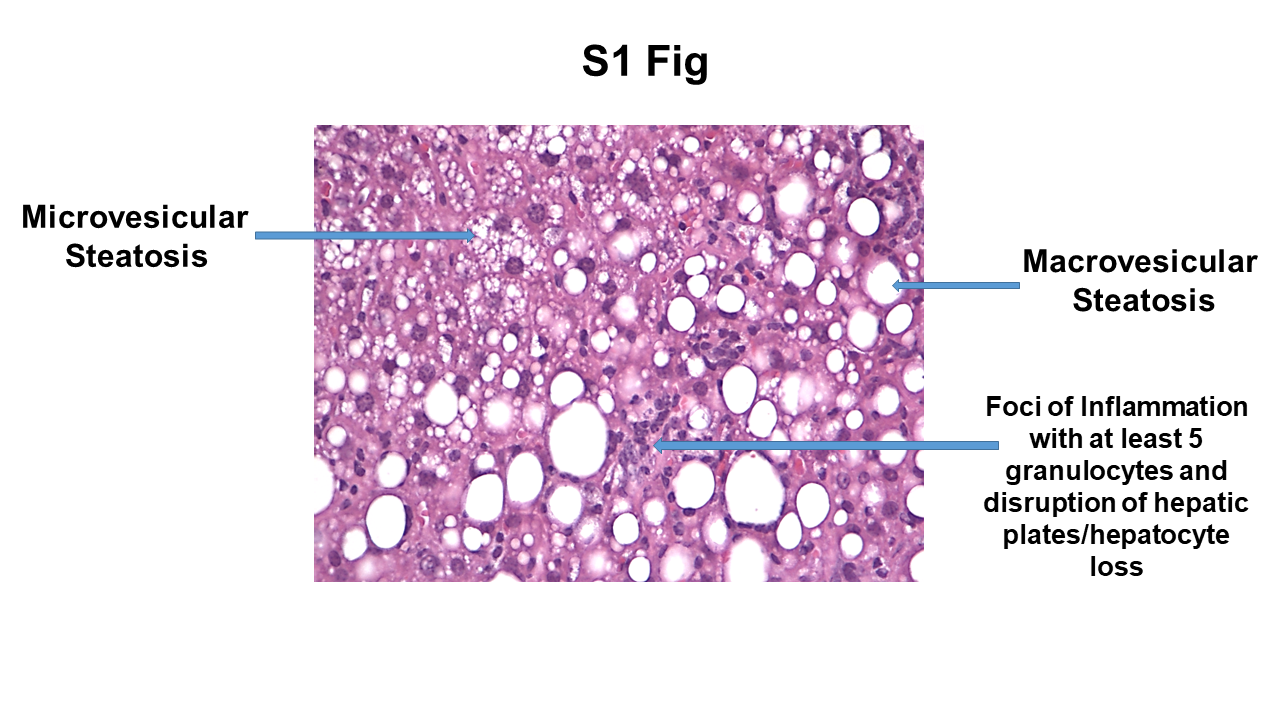

Supplement: S1 Fig — Liver of a WD-fed mouse (WD38) was fixed in buffered formalin, sectioned and stained with hematoxylin and eosin and photographed at 400x. Regions representing microsteatosis, macrosteatosis and inflammation, i.e., granulocytes, are marked. (TIF) [file pone.0214387.s001.TIF]

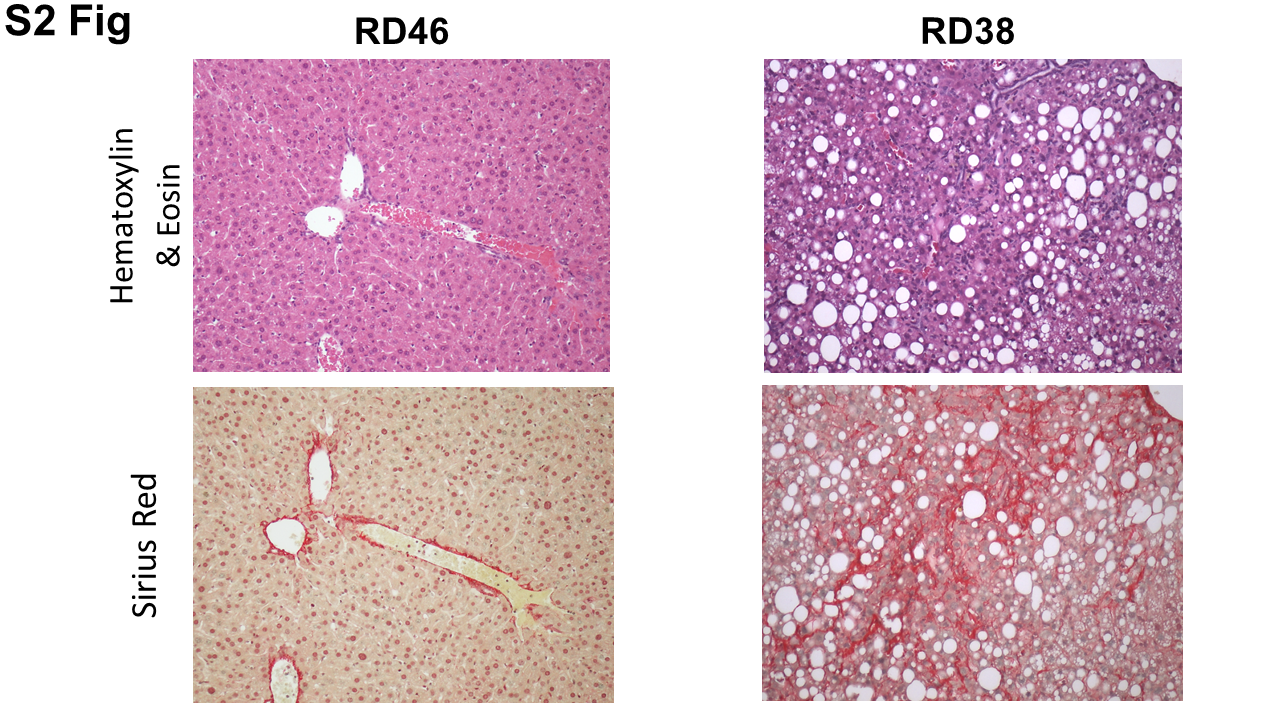

Supplement: S2 Fig — Livers of a control and a WD-fed mouse (WD38) were fixed in buffered formalin, sectioned and stained with hematoxylin and eosin and photographed at 200x. Liver from the control group (RD46) shows no signs of hepatosteatosis (H & E) or fibrosis (Sirius Red). Liver from the western diet group (WD38) shows extensive hepatosteatosis (H & E) and fibrosis (Sirius Red). (TIF) [file pone.0214387.s002.TIF]

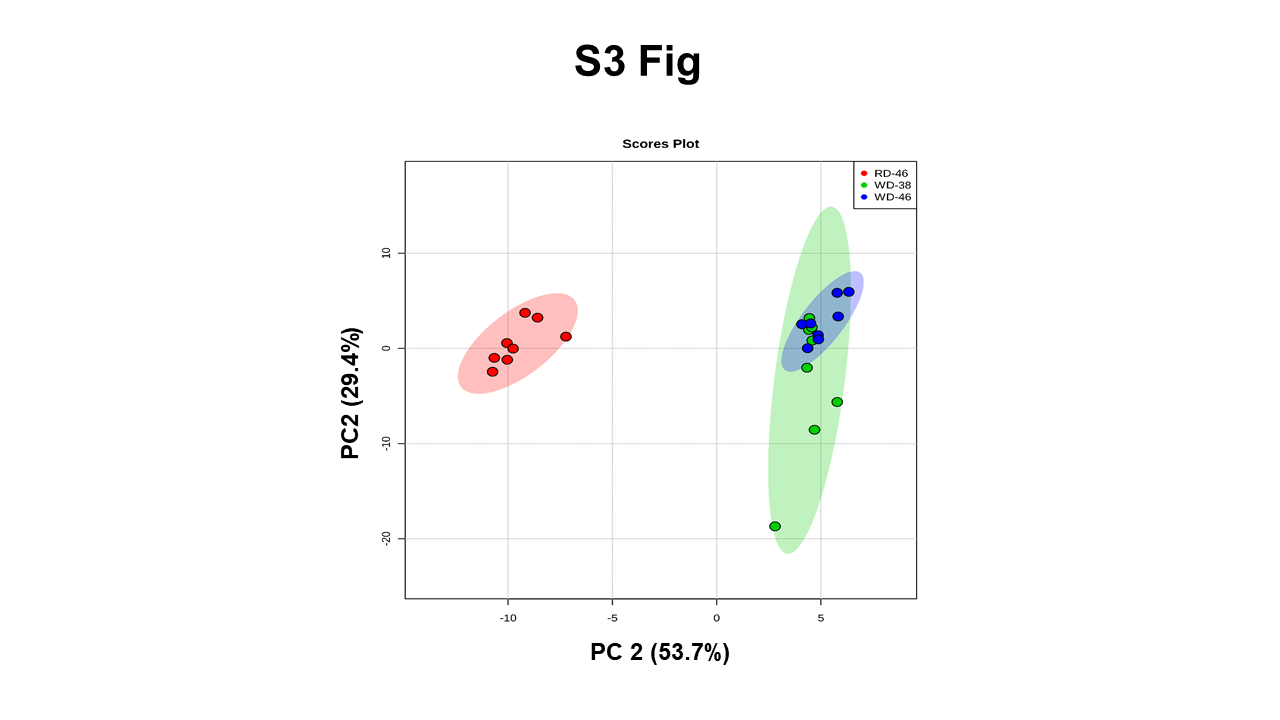

Supplement: S3 Fig — Data included in this analysis included only lipids quantified by LC/MS that were significantly affected by the WD. Liver lipidomic profiles differ depending on the diet and the duration of the diet. PCA was conducted after log transformation on all statistically significant lipids (q < 0.05) annotated in liver samples from female mice (positive and negative ion modes merged) and with a coefficient of variation < 30%. Each symbol represents an animal. N = 7–8 per group. (TIF) [file pone.0214387.s003.TIF]

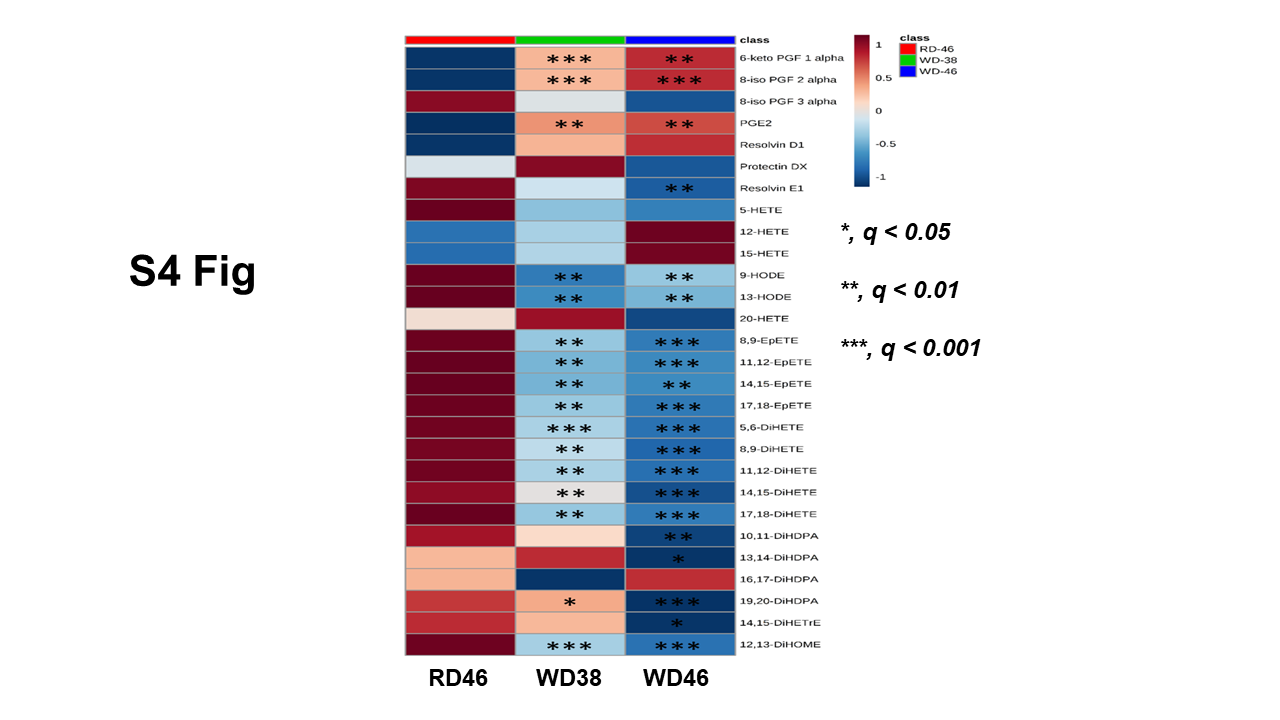

Supplement: S4 Fig — For each group the average value from 7–8 biological replicates was used. A logarithm data transformation and auto scaling (mean-centered divided by the standard deviation of each variable) were performed in MetaboAnalyst 4.0.in order to produce this visualization map. The represented q-values, accounting for differences between the treatments, were obtained as explain in the Materials and Methods section by using GraphPad Prism v7.03. (TIF) [file pone.0214387.s004.TIF]

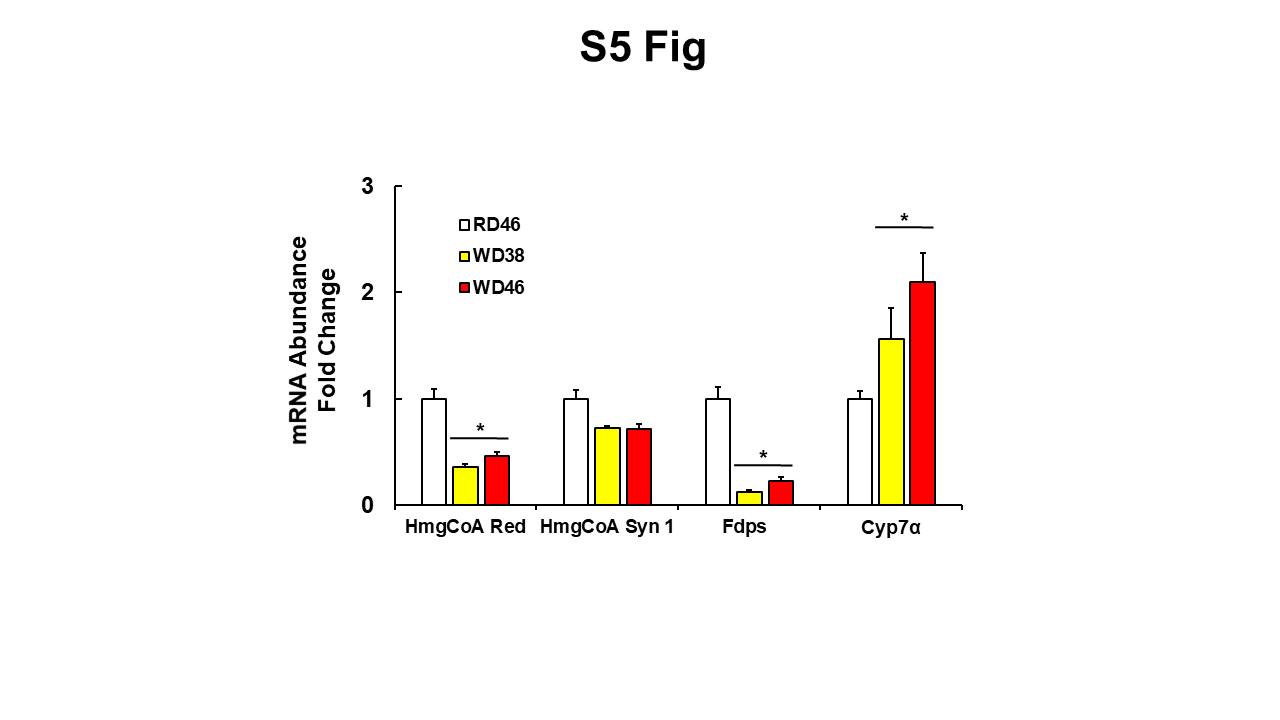

Supplement: S5 Fig — Transcript abundance was quantified as described in the Materials and Methods section. Results are expressed as mRNA abundance, Fold Change; mean ± SEM, N = 7–8 samples, *, FDR <0.05 versus the RD46 group. Hmg CoA Red: HMG CoA reductase; Hmg CoA Syn 1: Hmg CoA synthase 1; Fdps: Farnesyl diphosphate synthase; Cyp7α: Cytochrome P450 7α. (TIF) [file pone.0214387.s005.TIF]
