# Supplementary figures and images for: Correction: Lipidomic and transcriptomic analysis of western diet-induced nonalcoholic steatohepatitis (NASH) in female Ldlr -/- mice
Source: PLoS One. 2019 May 1;14(5):e0216535. doi: 10.1371/journal.pone.0216535 (PMC6493746; doi:10.1371/journal.pone.0216535)

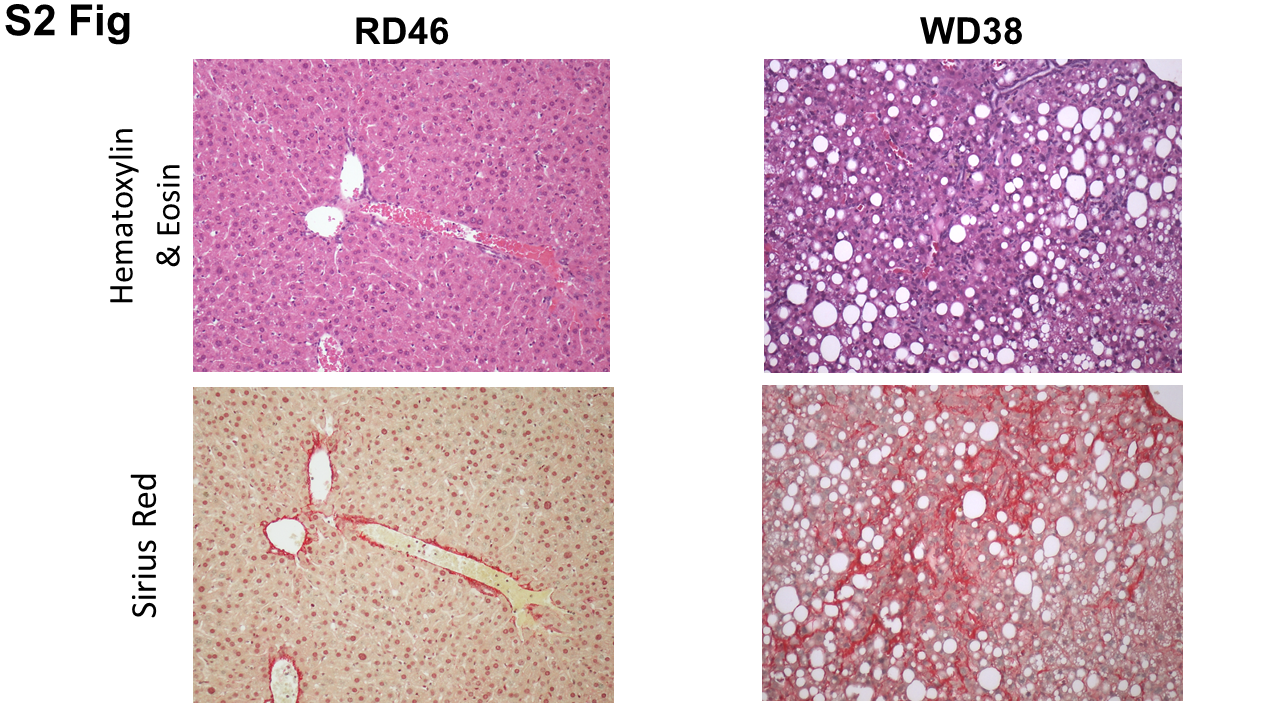

Supplement: S2 Fig — Livers of a control and a WD-fed mouse (WD38) were fixed in buffered formalin, sectioned and stained with hematoxylin and eosin and photographed at 200x. Liver from the control group (RD46) shows no signs of hepatosteatosis (H & E) or fibrosis (Sirius Red). Liver from the western diet group (WD38) shows extensive hepatosteatosis (H & E) and fibrosis (Sirius Red). (TIF) [file pone.0216535.s001.TIF]
